# On the influence of low-level visual features in film classification

**DOI:** 10.1371/journal.pone.0211406

**Published:** 2019-02-22

**Authors:** Federico Álvarez, Faustino Sánchez, Gustavo Hernández-Peñaloza, David Jiménez, José Manuel Menéndez, Guillermo Cisneros

**Affiliations:** Grupo de Aplicación de Telecomunicaciones Visuales, Escuela Técnica Superior de Ingenieros de Telecomunicación (IPTC), Universidad Politécnica de Madrid Avenida Complutense, Madrid, Spain; INSERM, FRANCE

## Abstract

**Background:**

In this paper we present a model of parameters to aesthetically characterize films using a multi-disciplinary approach: by combining film theory, visual low-level video descriptors (modeled in order to supply aesthetic information) and classification techniques using machine and deep learning.

**Methods:**

Four different tests have been developed, each for a different application, proving the model's usefulness. These applications are: aesthetic style clustering, prediction of production year, genre detection and influence on film popularity.

**Results:**

The results are compared against high-level information to determine the accuracy of the model to classify films without knowing such information previously. The main difference with other film characterization approaches is that we are able to isolate the influence of high-level descriptors to really understand the relevance of low-level features and, accordingly propose a useful set of low-level visual descriptors for that purpose. This model has been tested with a representative number of films to prove that it can be used for different applications.

## Introduction

Aesthetic film analysis has traditionally been an academic activity belonging to cinema studies, performed without using automatic tools. Nowadays manual tools such as Cinemetrics [1] provide an important help to film scholars, allowing for the extraction of metrics to compare styles from different films, filmmakers or aesthetic movements. However, key elements as shot changes have to be manually set, making it a tedious process, as well as inaccurate by the loss of meaningful information not perceptible by the human visual system.

A lot of applications are built around creative content, including recommender systems, decision support systems for marketing and distribution, customization and automatic generation. These tools and systems use semantic tags, collaborative models, or contextual characteristics, but they do not address issues regarding aesthetic characteristics of the content. This opens a potential opportunity for innovation as low-level aesthetic visual features can become new accurate parameters for characterization of films. How can visual features be useful to understand a film and how are they related to its production year or its genre? How can aesthetic film theories be used for improving the automatic classification of films? Probably, they have not been taken into account so far because of the limited automatic tools available to easily characterize an extensive set of films or due to the complexity of merging film theory, low-level video descriptors (using video processing) and classification techniques without considering high-level or semantic information. However, this is a necessary step to assess how relevant can low-level visual information can be by themselves. With this approach, the descriptors of both low and high levels can be separated and its influence understood. In this paper we focus on low-level visual features, and we verify a strong model which takes into account film theory allows for the automatic classification of films and the prediction of high-level attributes such as year of production or genre.

To do so, we have created a low-level visual descriptor model which can be used for aesthetically characterizing films. This has been tested using a representative number of films to prove that it can be used for different applications, for instance to characterize a video with unknown metadata or to support the classification of a film within a filmic movement or dominant film genre.

More specifically, the model is well-suited for the following applications: unsupervised classification (aesthetic style clustering), prediction of production year, genre detection and influence on the film popularity. Tests are developed for each of them and the model is proved effective for all uses. In addition, we demonstrated the suitability to combine with deep learning models (especially with convolutional neural networks) to improve the model results.

The paper is organized as follows. 2^nd^ section describes related work. 3^rd^ section explains the defined model of film characteristics and descriptors. 4^th^ section presents the tests and classification experiments performed to validate our model and system. Finally, 5^th^ section states both the future work and the conclusions. We included in an appendix the automatic extraction of descriptors explanation.

## Related work

### Film characterization and prediction techniques

The Internet and new technologies have made available new tools to characterize films. Cinemetrics [1], a crowdsourcing platform which allows users to make and insert specific metrics about films, has revolutionized film analysis. Thanks to the platform's tools and user collaboration, a big film metrics database has been created. However, this platform has several problems. First of all, it uses explicit information provided manually, which causes errors in some metrics, and contradictions among measures assigned to a single film by different users. Secondly, some important measures (mainly related to image and motion) cannot be quantified by the human eye and thus are not considered by Cinemetrics.

There are no automatic aesthetic video models completely comparable to our approach. However, previous works deal with some issues which are relevant for our objectives.

One of these issues is the influence of low-level video characteristics on users. Canini et al. [2] studied the affective influence of low-level characteristics, and we have relied on some of their findings and conclusions. They use some low-level characteristics which we have adopted in our model, in particular, those related to image features (light, color and saturation). However, they use only one descriptor to define the motion of a scene, and do not take into account syntactic or narrative pace. They classify scenes in three dimensions: cold/warm, slow/dynamic, minimal/energetic. They show that their descriptors do affect users. We expand this definition to other kinds of descriptors and apply it to full-length films (not only scenes). By creating a general aesthetic classification (not only related to users), we show that this expansion allows for the development of the new features described in this paper.

Canini bases his theoretical foundations on the interesting approach proposed by Wang and Cheong [3], who created a system that classified films into seven categories (anger, sadness, fear, joy/TA, surprise, neutral) using an audio descriptor and four visual low-level descriptors: shot length, motion, lighting and color energy. They group the descriptors into two dimensions, arousal and valence, and created the classification based on this emotional space. In our paper, we expand the set of descriptors, as will be explained in section “Film Characteristics and Descriptors model”, and apply them to characterize the entire film, not only scenes. Wang and Canini's reduced descriptor model is useful for the affective classification and recommendation of scenes, but additional descriptors are required to work with full films and use the model for other applications, as explained in section “Results”.

Another widely used method to classify both single scenes and complete films is the detection of audio events. Xu et al. [4] proposed the first approach to analyze scenes in horror and comedy films, expanded [5] using Wang and Cheong's theories about arousal and valence. Xu et al. [5] used Hidden Markov Models and Fuzzy clustering for the classification, achieving valuable results. Our classification tests also use a clustering algorithm to confirm the validity of our aesthetic model, but we focus only on visual descriptors.

Visual descriptors have been used in a variety of fields such as computational aesthetics or image indexing. The explosion in the use of aesthetic descriptors for video and images was started by Datta *et al*. [6], who used photography theory to model photograph rules. Our work uses video descriptors based on film theory, which opens new research possibilities. Concerning visual and aesthetic features, previous works such as [7] extract a number of features coming from emotion-based image recognition, computational aesthetics, and painting analysis. They predict an image's interest based on the system's training, using positive and negative ratings from Flicker images. This work, however, is oriented to images and not video and it does not follow the video descriptors based on film theory as we propose in this paper. Therefore, its results are not directly comparable.

In another work from the same authors [8], four features are used: 2 semantic features and 2 features for image appeal analysis (edge histogram), one proposed by the authors and the other taken from MPEG7 Edge Histogram Descriptors (EHD). This allows them to enhance the semantic analysis with high level information coming from composition analysis for scene recognition. Our aim is, however, to characterize films without using any semantic information and to focus on the characterization of films as we present in the paper.

In [9], a hierarchical approach is proposed to characterize the aesthetic appeal of consumer videos and automatically classify them into high or low aesthetic appeal. This approach uses 9 low-level features to characterize the aesthetic appeal of the videos and distinguishes between professional and amateurish videos. The purpose of the study is strictly limited to the foreseen application, as opposed to ours, which has multiple applications. Therefore, we need to integrate more than 9 low-level features for the automatic characterization of videos, considering all the applications we propose.

Other approach using visual descriptors is presented in [10] but limited to a total of 5 visual features, and in this case it is applied to video recommendation engines. Our model provides a wider range of visual descriptors and cover a broader application area.

In addition to the use of low-level descriptors extracted from video, other authors proposed to classify or characterize films using the video content (mainly video frames extracted from trailers) and deep learning techniques. This is the case of [11] and [12] where authors classify films using their trailers, with the application of Convolutional Neural Networks (CNNs), comparing the results with other deep learning techniques such as LSTM (Long short-term memory). Authors in [13] perform the classification with films poster images using CNNs, whereas authors in [14] are using previews. Applying deep learning but not to images can be found in [15] where authors use textual content from films databases with language neural models. The limitations of these techniques lie in the great computation time and heterogeneity required to compute the full films as we are doing in this work, and not only summaries, trailers, scenes, or even their advertisement material as the papers mentioned above, besides the difficulty in understanding and characterizing a full film, with evolving content and different kind of scenes.

### Film theory

Film theory is an important research field that began with the start of the 20^th^ century and reached its maturity throughout the years. After photography and cinema were invented in the 20^th^ century, the ideas of German philosopher Walter Benjamin about the “Mechanical Reproduction” of Art [16], changed the art analysis and representation paradigm. These ideas have recently re-emerged with the digitization of photography and cinema, which allows a complete and exact reproduction of the original object. The work of art is not a physical object anymore; artistic properties are included in the logic, in the information. This opens a stimulating way in automatic analysis of visual features, and art theories should be taken into account for that purpose. In this paper, we focus on automatic classification of films, considering visual features from an aesthetic point of view.

Since the times of silent films, several technical characteristics were extracted by some formalist film theorists, such as Sergei Eisenstein [17] or Béla Balázs [18], both of whom defined editing and visual language features. However, the most complete theoretical formalization was developed by Jacques Aumont et al. [19], and it is still the main reference in audiovisual communication nowadays.

The taxonomy proposed by Aumont et al. is taken as a reference of general characteristics, whose modeling for our approach is explained in section “Film Characteristics and Descriptors model”.

A more specific approach to some general categories (specially about narrative and visual influence of editing) have been inspired by the two most important current film theorists in this area, Noël Burch [20] and David Bordwell [21].

Besides, these studies have been completed with other approaches regarding the sensory experience evoked by images (Gombrich [22]) as well as human influence and computation of color (Itten [23] and Davis [24]). We already took into account these approaches when working with pictures for still image characterization, which we developed in previous research works [25].

Another important issue for the paper is to create a ground truth to validate the results. To this end, it is important to classify each film of the data set, in a manual way, according to its aesthetic style (to validate the test in sub-section “Unsupervised classification: aesthetic style clustering”) and its genre (to validate the tests in 5.3). The aesthetic styles used as reference have been obtained from David Bordwell’s canonical book “The Classical Hollywood Cinema: Film Style & Mode of Production to 1960” [26], Adrian Martin’s “What is Modern Cinema?” [27] and Thanouli’s “Post-Classical Cinema: An International Poetics of Film Narration” [28]. They are: “Silent films”, “Classic films”, “Modern Films”, “Neoclassical films” and “Postmodern films”.

A thorough study of the film theory literature was carried out to assign each film to a specific category. The distinction between “Classic films” and “Modern films” was performed using especially David Bordwell’s aforementioned book [26] and James Chapman’s film history review [29]. The distinction between “Neoclassical films” and “Postmodern films” was made using Thanouli’s book about post-Classical Cinema [28]. A thorough study of film theory is essential for our aim, because the expansion of previous research works, such as the ones proposed by Wang [3] or Canini [2], is based on this aesthetic approach.

## Film characteristics and descriptors model

Our model uses low-level descriptors to automatically characterize films according to aesthetic criteria. It also allows us to predict high-level aesthetic features and to classify films according to visual style (which is normally done by analysts). It provides other functionalities, such as the prediction of production year.

To create such aesthetic model, two separate knowledge fields, film theory and automatic video processing, are mixed.

The main visual characteristics, taken from film theory by Aumont et al. [19], are classified into three main categories: image, pace and motion. In each category, a set of 24 descriptors is defined. The selection of descriptors was done by pruning a preliminary list of 30+ descriptors.

To select relevant descriptors we did an analysis of the potential influence of the descriptors in how users perceive films (which can be linked to applications such as content recommendation, popularity prediction, classification…) and we took a relevant database such as MovieLens [30] to prune a long list of descriptors, which can useful for full films, not only to scenes or trailers. In the case of applying only to selected scenes, the results obtained may not be the same.

We used a known dataset, MovieLens10M, with more than 10000 films, 71567 users and 10 million ratings to select a reasonable number of descriptors for our work.

The first step was to extract the descriptors, and normalise their values with a proportion considering the elements range.

To select a meaningful number, we used the Kolmogorov-Smirnov method, calculating the value of the influence with respect to the users ratings. We selected users with at least 10 rated films, resulting in testing against 30109 users preferences.

**Fig 1:**
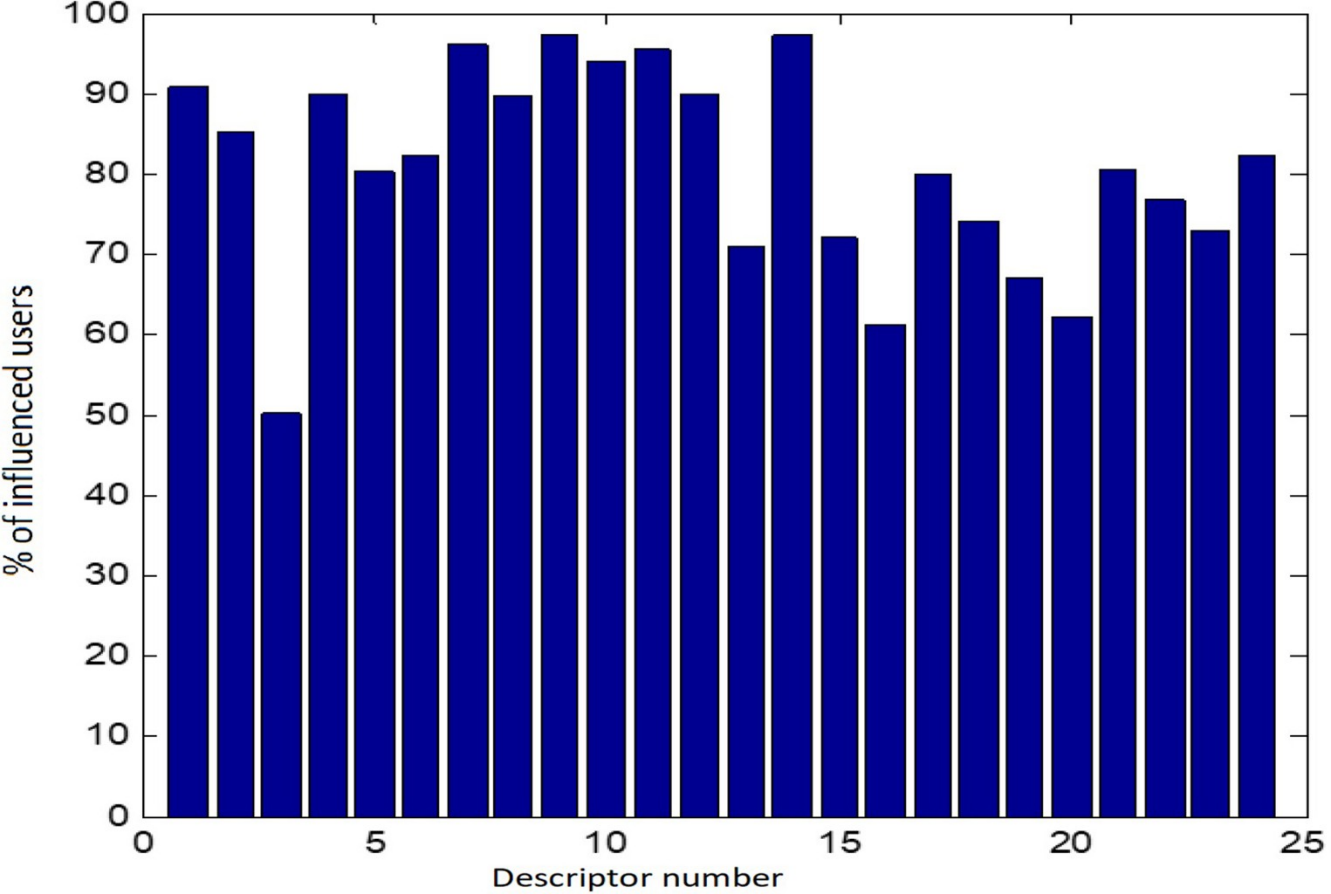
Influence of each descriptor, using Kolmogorov-Smirnov test.

We used a significance value of α = 0.1, and we selected the descriptors which have an influence to at least 50% of the users. The results can be found in Fig 1

The results for other descriptors are below 50%, so we consider them as not relevant for our study.

The discarded ones were either not useful to classify films (after the tests carried out and explained in section “Results”) or not automatically detachable with sufficient quality (for example, descriptors related to visual geometric composition and harmony). One interesting case are some complex visual descriptors which were taken into account, such as Histogram of Oriented Gradients (HOG), Motion Boundary Histogram (MBH) or dense trajectories, but after checking their relevance through statistical tests (Kolmogorov-Smirnov and Mann-Whitney) they did not reveal useful, because they don’t provide valuable information that is not represented through the simpler descriptors. The reason why these descriptors can be used in scene understanding applications but not in this case, might be in the different scenario we are facing: we are using full films which are composed by many heterogenous scenes as well as a broad range of applications we are proposing in this paper.

The final descriptors of the model are specified below:

1) Image (6 descriptors)

Image descriptors characterize features related to brightness, color and composition. The values of these attributes are usually homogeneous inside a shot, and only need to be processed in the key frames, saving processing power and time. A homogeneity threshold needs to be applied: if the threshold is met, the mean value of the shot frames is taken.

Our model's descriptors in this first block are based on previous works of the authors [25][31], where their effectiveness has been proved. These descriptors, as it has been underlined, are based in Aumont's film studies (focused on aesthetic studies), although some of them are also similar to the technical descriptors defined in MPEG-7 [32][26]. The descriptors have been refined and some details have been adapted from the “still images” scenario to the “video” scenario.

Three descriptors are directly related to the brightness of the film:

- *Black and white rate* (BWR) It determines how many shots are filmed in black and white or in color, and it is expressed by (1), where T is the total length of the film and T_Bw_ is the length of the shots that the system classifies as black and white shots.

$$BWR=\frac{T_{BW}}{T}$$ (1)

- *Luminosity* (average, LUM, and variance, LUV). These two descriptors are used to characterize the general brightness of the film. The value of the luminosity has been taken directly from the Value of the Hue, Saturation, Value (HSV) color space. It has been checked (with the 200 complete films used in our corpus) that luminance distribution values are Gaussian. Therefore, mean and variance are the metrics used for the characterization. In (2) and (3), L_mk_ is the mean luminance of the pixels of the key frame selected for shot k. K is the total number of shots in the film.

$$LUM=L_{AVE}=\frac{1}{K}\sum_{k=1}^{K}L_{mk}$$ (2)

$$LUV=\frac{1}{K}\sum_{k=1}^{K}{\left(L_{mk}-L_{AVE}\right)}^{2}$$ (3)

The next two descriptors are related to the color features of the films:

- *Saturation* (SAT). Saturation is one of the main concepts in colorimetry, and it is taken directly from the S component of HSV. S_m_ is the mean saturation of the key frame of the shot k.

$$SAT=S_{AVE}=\frac{1}{K}\sum_{k=1}^{K}S_{m}$$ (4)

- *Chromatic variety* (VC) This descriptor is related to color and composition features, providing an approximation for the distribution of forms in the structure of the image. Chromatic variety represents the structure of the color in the image. The chromatic variety of an image is given by VC_i_, which is defined as the variance of the bins of the total color histogram for the image, where N is the total number of bins and $B_{n}$ is the value of the bin n.

$$VC_{i}=\frac{1}{N}\sum_{n=1}^{N}N\cdot {\left(B_{n}-B_{AVE}\right)}^{2}$$ (5)

We finally take the average of ${VC}_{i}$ as the descriptor for chromatic variety:

$$VC_{AVE}=\frac{1}{K}\sum_{k=1}^{K}VC_{i}$$ (6)

The last descriptor of this block is also related to composition:

- *Entropy of luminosity* (ENM). This descriptor represents the amount of information contained in an image. It can be used, for example, to distinguish shots of different kinds (long shot, American shot, close up…). It is calculated using the usual expression of entropy, applied to the quantified levels of grey (L) in each pixel (i) for each shot (k) (7).

$$ENM=\frac{1}{K}\sum_{k=1}^{K}\sum_{i}^{N_{P}}\left(-P\left(L_{ki}\right)\cdot \log_{2}L_{ki}\right)$$ (7)

2) Pace (10 descriptors)

Pace descriptors are related to the syntax and narrative of the visual content. They provide an approach to the amount of visual signs used to communicate information. Aumont et al. [19] define three complementary functions in the editing of an audiovisual piece of content, namely syntactic, rhythmical and semantic functions.

Syntactic and rhythmical functions can be modeled by taking into account some objective metrics, which are integrated in our approach. These metrics are based on the three elements of film editing: cut, fade and dissolve. In contrast, semantic function is not only related to signifier, but also to meaning, which makes its automatic processing based on low-level features more difficult. However, a basic approach to this function is obtained using metrics over scenes, and connecting syntactic metrics (shots) with scene metrics. It is a low semantic level, but it is useful as a description of how visual aesthetics interfere/correlate with semantic fields. Semantics is out of scope of this research, but we deal with the influence of aesthetics in the semantic function.

Ten descriptors have been defined in this group, taking into account the aforementioned theories and features:

- *Average Shot Length* (ASL) and *Variance of Shot Length* (VSL). These two descriptors are used to characterize the length of the shots of a film. Both descriptors are used by film scholars [20] [21] in their research about film styles (e.g. using the manual tool Cinemetrics [1]). *S*_*n*_ is the time between any of these events: cuts, fades, dissolves. The event detection processes are explained in section “Automatic Descriptors Extraction”.

$$ASL=S_{AVE}=\frac{1}{K}\sum_{n=1}^{K}S_{n}$$ (8)

$$VSL=\sqrt{\frac{1}{K}\sum_{n=1}^{K}{\left(S_{n}-S_{AVE}\right)}^{2}}$$ (9)

- *Median Shot Length* (MSL). In certain filmic styles, the mean is not an accurate parameter to characterize the length of the shots, and the median has to be used. Besides, sometimes the relation between mean and median is useful to characterize some visual styles, as this helps to obtain skewness and kurtosis factors of the statistical distributions. MSL is given by the median value of shot length.

- *Fade Rate* (*FAR*) and *Dissolve Rate* (*DIR*). Fade Rate expresses the rate of shots ending with a fade out or beginning with a fade in. The expression for DIR descriptor is analogous for dissolve events. They are defined in (10), where *F* is the number of fade events detected, D is the number of dissolve events detected, and *K* the total number of shots of the film.

$$FAR=\frac{F}{K-1};DIR=\frac{D}{K-1}$$ (10)

The rest of descriptors are analogous to the ones defined but applied to scenes. These descriptors are: *Average Scene Length* (*ACL*), *Variance of Scene Length* (*VCL*), *Average Shot/scene Rate* (*ASR*) and *Variance of Shot/scene Rate* (*VSR*). The last descriptor is obvious but useful: *Runtime* (*RUN*), which expresses the total length of the film, in minutes.

3) Motion (8 descriptors)

Two kinds of motion can be distinguished: camera motion, which results in the homogeneous motion of the image (caused by the motion of the camera); and the internal motion in the image (assuming a static camera, the movement of people or/and objects). Camera motion and internal motion generate different stimuli to the observer. Therefore, they should be modeled as different descriptors. A good example to illustrate this need is given by people who dislike “Dogma” films [33] [26], or other hand-held shot films (because it bothers them), but they like other still shot films with similar aesthetics. It is also important to distinguish between intensity and complexity. Following the example, some people who do not like hand-held shot films (an irregular, complex kind of motion) like films with similar or higher intensity of motion, but with harmonious motion (shot in pans or tracking shots). Internal and camera motion are valuable descriptors to characterize different aesthetic styles.

The procedure for obtaining these descriptors is to measure the optical flow of several points, external and internal which are homogeneously distributed in each frame. The implementation of this procedure in explained in the optical flow section in the S1 File.

Descriptors in this block are described below:

- *Camera Motion Intensity* (CMI). When camera motion is detected, most of the pixels in the image are moving homogeneously. Therefore, in each frame, the external pixel with the median optical flow in frame w (pixel *i*) is selected to represent camera motion (thus avoiding internal movement, objects and people). Its optical flow, in Cartesian coordinates, is represented by x_i_,_w_, y_i_,_w_. MIn is obtained for the *Q* frames in a shot, and *CMI* is obtained as the mean value of the *K* shots in the film.

$$MI_{n}=\frac{1}{Q}\sum_{w=1}^{Q}\sqrt{x_{i,w}^{2}+y_{i,w}^{2}}$$ (11)

$$CMI=\frac{1}{K}\sum_{n=1}^{K}MI_{n}$$ (12)

- *Camera Motion Complexity* (CMC). As with *CMI*, median pixel optical flow is chosen in each frame, but now the orientation histogram *h(n)* along a shot is taken into account. The possible orientations considered are 0º, 45, 90º, 135º, 180º, 225º, 270º and 315º (bins b). The entropy of the median pixels' orientations in a shot *n* (one pixel per frame *w*) is calculated as follows.

$$CMC=\frac{1}{K}\sum_{n=1}^{K}\sum_{b=1}^{K}h_{b}\left(n\right)Log(h_{b}\left(n\right))$$ (13)

-*Internal Motion Intensity* (IMI). Obtained by subtracting the camera motion of each shot, MI_n_ (which can be directly measured) from the total motion of each shot, MV_n,_ which is measured from the optical flow obtained using internal and external points in each frame. The IMI of a film is then given by the average value of its K shots.

$$IMI=\frac{1}{K}\sum_{n=1}^{K}\left(MV_{n}-MI_{n}\right)$$ (14)

-*Internal Motion Complexity* (IMC), *Camera Motion Intensity Variance* (CIV), *Camera Motion Complexity Variance* (CCV), *Internal Motion Intensity Variance* (IIV), *Internal Motion Complexity Variance* (ICV) are modeled analogously to the descriptors already defined. Descriptors in this block have been modeled using mean and variance because they fit a Gaussian distribution.

The final list of descriptors and corresponding identifiers is presented in Table 1.

**Table 1:** Final list of descriptors.

Id	Descriptor name	Descriptor type
1	Black and White Rate (BWR)	Image
2	Mean of Luminosity (LUM)	Image
3	Variance of Luminosity (LUV)	Image
4	Saturation (SAT)	Image
5	Chromatic Variety (CRV)	Image
6	Mean of entropy (ENM)	Image
7	Average Shot Length (ASL)	Pace
8	Variance of Shot Length (VSL)	Pace
9	Median Shot Length (MSL)	Pace
10	Runtime (RUN)	Pace
11	Average Scene Length (ACL)	Pace
12	Variance of Scene Length (VCL)	Pace
13	Average Shot/scene Rate (ASR)	Pace
14	Variance of Shot/scene Rate (VSR)	Pace
15	Fade Rate (FAR)	Pace
16	Dissolve Rate (DIR)	Pace
17	Camera Motion Intensity (CMI)	Motion
18	Camera Motion Complexity (CMC)	Motion
19	Internal Motion Intensity (IMI)	Motion
20	Internal Motion Complexity (IMC)	Motion
21	Camera Motion Intensity Variance (CIV)	Motion
22	Camera Motion Complexity Variance (CCV)	Motion
23	Internal Motion Intensity Variance (IIV)	Motion
24	Internal Motion Complexity Variance (ICV)	Motion

## Testing and results

Some classification tests have been developed to validate our aesthetic film model. We present two kinds of tests: the first kind of tests is an unsupervised classification, which we use to validate the correct clustering of films according to their style (“aesthetic style clustering”), as we have defined in section “Related work”; the second kind of tests is a supervised classification, which we use to predict high-level features: production year and genre tests, which are theoretically independent from our descriptors and a last test was done to assess the influence of the descriptors model in the film popularity. The unsupervised classification test is focused on the precision and recall of the aesthetic style classification, and the purpose of the supervised tests is to check the possibilities of our approach in order to predict quantifiable high-level features, which are related, in an indirect way, to the aesthetic of films.

Before the explanation of the tests, a description of the used dataset is needed.

### Dataset and framework description

A set of films was used for validating the system. The films were selected from the same well-known film database explained above, MovieLens 10M [30], containing 10,000,054 ratings, 10,681 films and 71,567 users, and in the future it will allow combining our aesthetic model with user preferences (rating) to create new applications. However, these data are not enough to directly apply our model, because we need to process the entire film for its aesthetic characterization. Therefore, we took a subset of some DVD films available in the MovieLens dataset. A set of 200 films was chosen considering that diverse film styles should be represented. To this end, films from several decades (from year 1920 to 2008) and a variety of countries (14 in total) were selected. Popularity criteria were also considered, as this is a good way to have a heterogeneous data set. Thus, the number of MovieLens ratings was taken into account when selecting the films. The minimum percentage criteria and the final number of selected films are shown in Table 2.

**Table 2:** Distribution of films by rating.

Number of ratings	Minimum number of films	Real number of selected films
>10000	10% (20 films)	16.5% (33 films)
5001–10000	15% (30 films)	17.5% (35 films)
501–5000	25% (50 films)	30% (60 films)
51–500	15% (30 films)	24% (48 films)
1–50	10% (20 films)	12% (24 films)

Diversity of the production year was also taken into account for the selection (this is detailed in the prediction of production year test), as it enables to perform the year prediction test and it ensures the presence of a greater variety of styles.

In order to make correct access to the data easier, the films are processed by our automatic tool, and the extracted descriptors from the 200 films are inserted into a MySQL database. 200 films have been chosen because they are enough to limit the sampling error (explained in sub-section “High-level characteristics prediction”). These data are then normalized using the Z-score method (15), as it preserves the ranges and dispersion of the distributions. The parameter x represents the absolute value, μ the mean of the distribution, σ the standard deviation, and z the normalized value. The normalization stage allows to mix the descriptors in the next classification tasks.

$$z=\frac{x-\mu }{\sigma }$$ (15)

**Fig 2:**
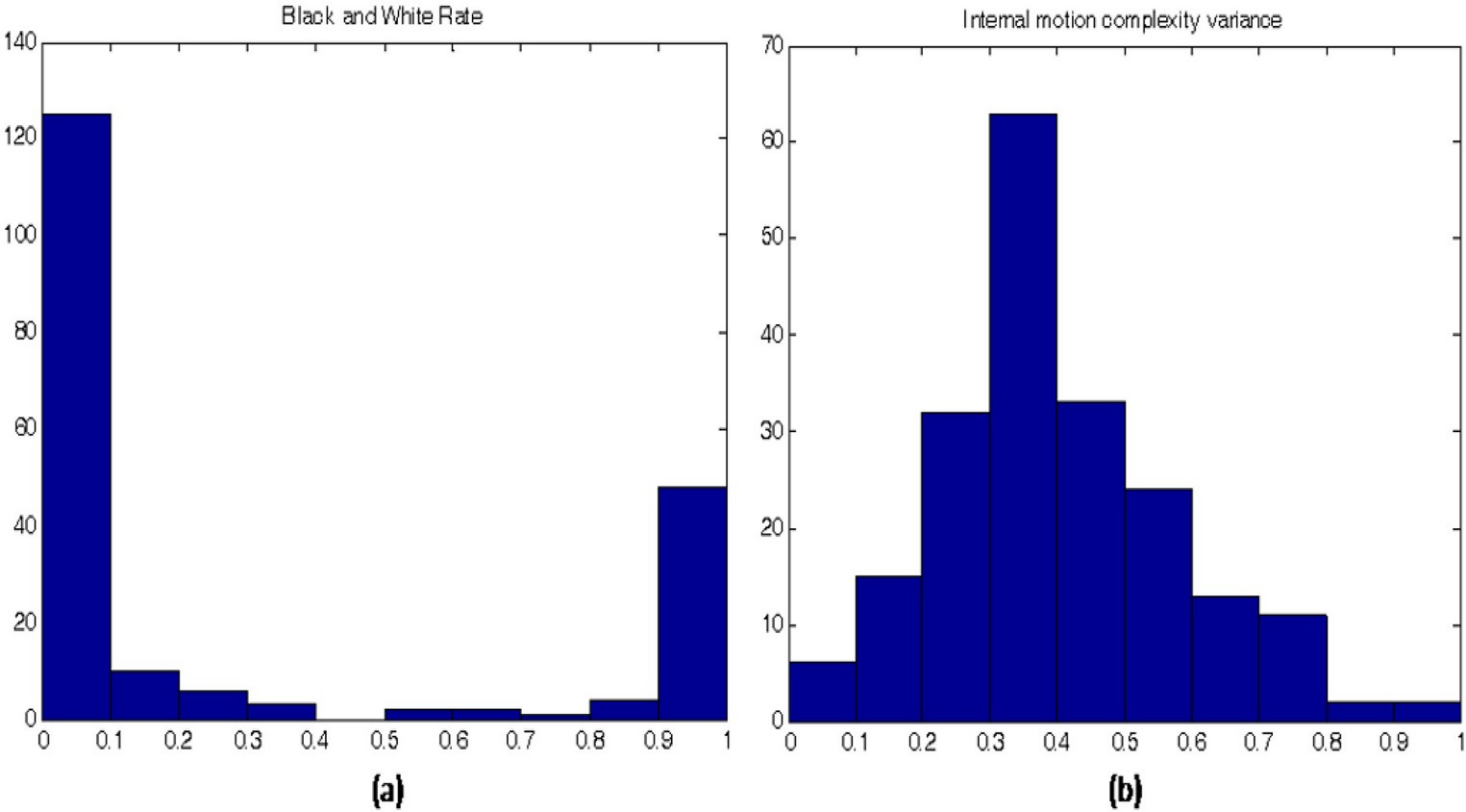
Comparison of Histogram descriptors.

Another important feature to be considered is that each descriptor has a different statistical distribution, as depicted in Fig 2, which compares two of these descriptors: Black and White Rate (a) and Internal Motion Complexity Variance (b).

In addition to our dataset and to allow comparison with other works in the application to perform genre classification, we used as well the dataset released in [15] (named Large Movie Training Dataset or LMTD from now on) for the training process. This dataset is composed of 2875 films, classified into 9 genres.

### Testing: Experiments and rationale

In order to assess the performance of our model, we developed different tests, using the dataset with 200 full films. The tests aim to cover different applications, based on classification or prediction of results, to demonstrate the versatility and applicability of our solution only using low-level image features. Depending on the application, we use supervised or unsupervised learning models (for classification or categorization, and regression we used supervised, for classification into clusters and association we used unsupervised).

We developed a total of 4 experiments, which we believe it can give a good overview of the applicability of our approach.

- We performed a test on the capability of our descriptors model to correctly cluster films into aesthetic styles, using unsupervised learning (Test 1).
- We have performed two tests on the effectiveness of the model to predict high-level characteristics, using objective data set up in advance. These tests are based on a supervised classification. Both tests classify objective high-level features of films, namely, production year (Test 2) and genre (Test 3). Different learning schemes have been developed and compared with current practices in the last years, with a combination of low-level descriptors and spatial and temporal image features, using deep learning approaches.
- We developed a last test to assess the potential influence of the descriptors in the prediction of the popularity of a film (Test 4), using a supervised learning model.

#### Test 1: Aesthetic style clustering

The purpose of the first test is to prove the use of the descriptors to cluster the films into predefined aesthetic clusters, where a film style classification can be made over a set of films without using any kind of training.

The first test is developed using an unsupervised classification technique: the clustering of films. The defined aesthetic features are good enough to classify aesthetic styles or tendencies.

**Fig 3:**
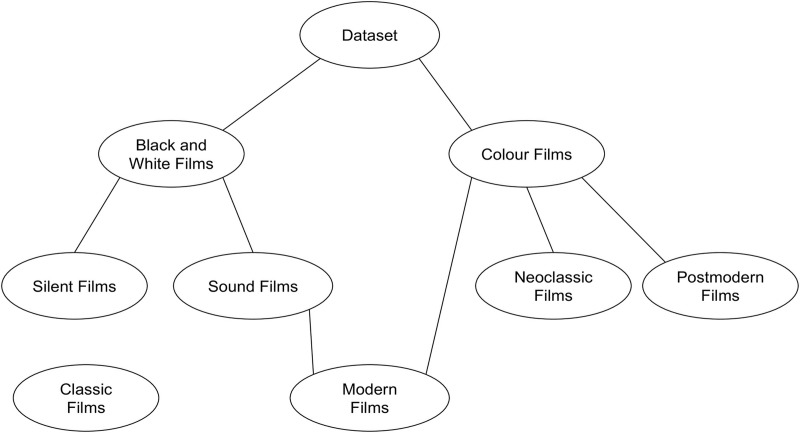
Hierarchical clustering, where final nodes are the selected groups for classification.

The test has been developed over the global dataset of 200 films described in section “Dataset and Framework Description”. First, the films were classified manually into their corresponding aesthetic group, as it has been explained in sub-section “Film theory”. Fig 3 presents the hierarchical aesthetic style clustering, which is based on the studies done by authors in [26][27][28]. We used the final nodes as the groups selected for the classification.

The total number of films in each group is detailed in Table 3. The sampling error [34] is also calculated for a sample of 200 films in an infinite universe (>100,000), for a 95% confidence interval (sampling bias occurs when a sample statistic does not accurately reflect the true value of the parameter in the target population). The obtained sampling errors have been studied to set the final number of 200 films and the limit of the sampling error in our scenario had been set to 7%.

**Table 3:** Films of dataset and chosen groups.

Film Aesthetic Group	Number of films	Sampling error
Silent films	11	3.16%
Classic BW films	33	5.14%
Modern films	13	3.42%
Neoclassic films	97	6.93%
Postmodern films	46	5.83%

Aesthetic style classification is made by means of hierarchical clustering, where sound black and white films can be divided into classic or modern films (pre-modern films, or independent films in the context of classical cinema have been also included in this category), and color films can be mainly divided into neoclassic or postmodern (blockbusters and independent postmodern) films (see Fig 3). However, some outliers of color films have been classified as modern films too (mainly European or Asian films of the 60’s or 70’s, previous to postmodern age [27]).

The films in the dataset are described only by the 24 aesthetic descriptors defined in Table 1, and the descriptors are normalized as explained in sub-section “Dataset and Framework Description”. Before starting the clustering, a previous Principal Component Analysis (PCA) was performed on the films. The number of final chosen components corresponds to the amount of information of the eigenvectors, and a value of 80% of the total information is chosen to remove the noise from the other components. In the performed tests, 6 eigenvectors are needed to obtain 80% of the global information.

Therefore, the clustering is performed over the 200 films in a 6-dimensional space. A more homogeneous or heterogeneous dataset will change the number of selected dimensions, because the amount of information should remain constant.

#### Aesthetic style clustering results

The implemented clustering divides each group into two new groups, which correspond to the groups defined in Table 3.

The clustering process uses a k-means algorithm, with a Euclidean distance to compute the measures. The precision and recall results of each class are shown in Table 4.

**Table 4:** Results of the classification.

Film Aesthetic Group	Precision	Recall
Silent films	77.8%	63.6%
Classic BW films	79%	91%
Modern films	80%	61.5%
Neoclassic films	90.8%	91.8%
Postmodern films	82.2%	80.4%

It can be seen that the classification for “Colour Films” (as presented in Fig 3) presents very high performance results. For “Black and White Films” (see clusters in Fig 3 results are also good, the worst results being for the recall measure of “Silent Films” and “Independent/modern BW Films”. This is because some films are on the fuzzy borders between “Silent Films” and “Classic BW Films” or between “Independent/modern BW Films” and “Classic BW Films”. Besides, the first sound movies in History showed a visual style very similar to silent films, given that sound was incorporated into films abruptly, while visual style had yet to evolve. Another important feature which explains this lower result in these two categories (as we can see in Table 4) is that they have fewer items and its associated clusters present a lower density. This means that the centroids of these clusters are less bold than centroids associated to categories with more elements.

#### Test 2: Prediction of production year with two approaches

The visual aesthetics of a film depends on several factors, including contextual factors such as the production year. The general aesthetics of films has changed throughout film history. Therefore, as we can classify different visual styles, then we are able to approximate a film's production year.

The oldest film of the dataset is from 1920, while the newest was made in 2008; therefore, the dataset covers 88 years of History. The distribution of films by decade is shown in Table 5.

**Table 5:** Distribution of films of the dataset by decade.

Decade	20	30	40	50	60	70	80	90	00
Number of films	9	8	15	15	24	13	12	55	49
Sampling error (%)	2,9	2,7	3,6	3,6	4,5	3,4	3,3	6,2	5,9

A multiple regression technique is used to predict the production year. One set of films is separated from the others and used as training set. The quality of the prediction is measured using the Mean Absolute Error (MAE), which computes the mean error of each predicted film year.

In the first approach we used a forward stepwise regression.

In this case, not all descriptors in Table 1 are useful. We have created a brute-force algorithm following a forward stepwise regression [35] to select the important descriptors for the year prediction function. The 10 descriptors used, using the IDs in Table 1, are: 1, 2, 6, 8, 9, 10, 11, 12, 14, 15, 18, 19, 20, 21, 22, 23 and 24. Overfitting problems were avoided using Lasso regularization [36].

The year prediction capability of the model is validated using a Repeated random sub-sampling validation, where it has been checked that it converges from 5,000 folds on, for any training set size (i.e., when the training set comprises 120 films, there are $\left(\begin{array}{c} 200\\ 120 \end{array}\right)=$ 1.64· 10^57^ possible combinations). In each iteration of the model, training and testing sets are chosen randomly.

**Fig 4:**
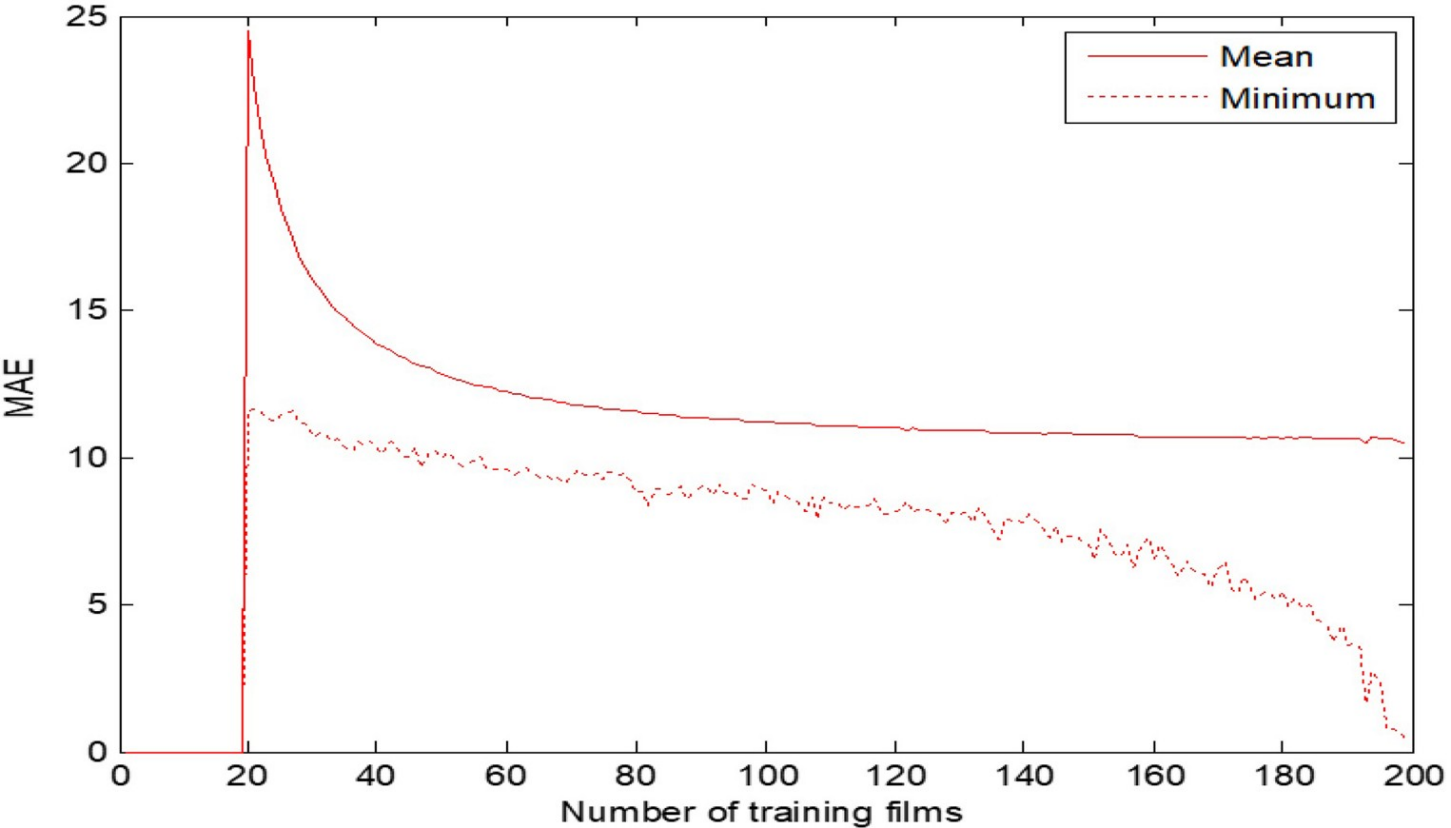
MAE of production year prediction test.

Several tests (with different rates of training/testing films) have been carried out. The performance of the model can be seen in Fig 4 and Table 6, where a Repeated random sub-sampling validation (k = 10000 folds) has been run.

**Table 6:** Production year prediction results.

Number of training/test films	40/160	80/120	120/80	160/40
MAE mean (k = 10000)	13.88	11.54	10.99	10.72
MAE min	10.28	9.11	8.38	7.02

For training sets higher than 160 films, the performance remains almost constant. We can see that, in this case, the model can predict the production year of a film with a mean error of only 10.72 years.

A better result would be very difficult to reach, because visual film styles do not change very fast, and films of the same decade present a very similar aspect. Besides, it is difficult to classify properly some films which imitate styles from other periods. Nevertheless, we have checked that the MAE can be reduced to less than 10 years by building manually the training set with representative films from each period of time. This can be seen in MAE min, in Table 6, which shows the result for the optimal training set.

Since there are a good number of films from similar years, we calculated the Interrater reliability by using the Kappa statistic. Both approaches in this test results have been compared using Kappa Score [37].

In this approach the results showed a K = 0.619, coming from ρ_o_ = 0.724 ρ_e_ = 0.276, which can be considered as good.

In the second case, instead of using a forward stepwise regression as presented in the former result, we added as learning scheme an artificial neural network.

We have designed the network with eight hidden layers, after testing different number of layers, it presented the most consistent result. The training selected is done by using Bayesian regularization. This algorithm typically requires more time to train, but can result in good generalization for difficult, small or noisy datasets. Training has been done using 70% of the dataset (140 films) while validation and testing used 15% each (30 films).

**Fig 5:**
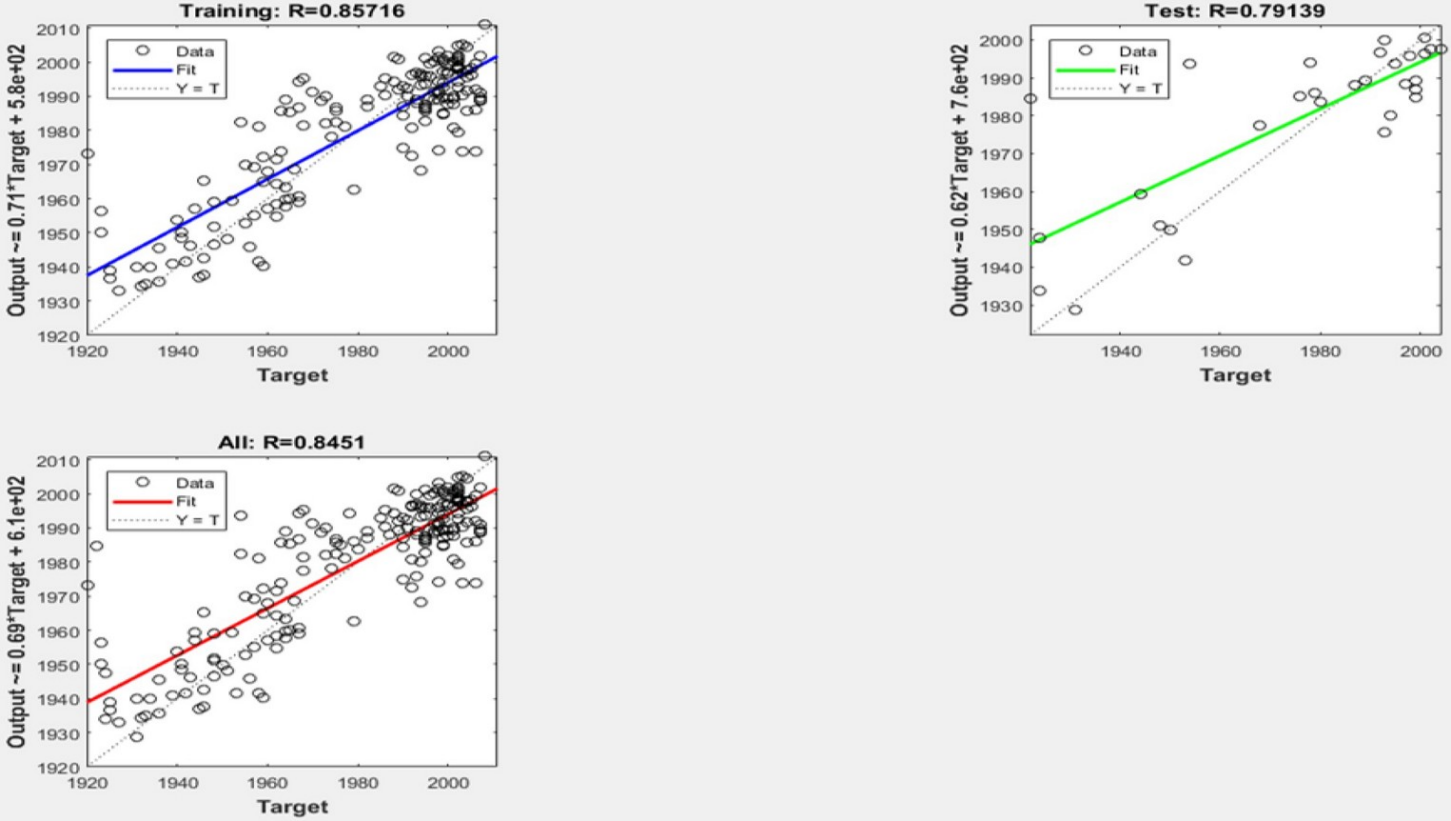
Results of the year prediction using a neural network.

Results from the neural network application for production year prediction (with 100 epoch) are RTrain = 0.86 / RTest = 0.79 / RTot = 0.85 as it can be found in the Fig 5.

As shown in the Fig 5 the unbalanced distribution of the films leads to accuracy problems in the early decades of the 20th century. But most of the results predicted are assigned to the same decade of the real production. In terms of interrater reliability we used the Kappa statistic, in a similar manner as in the last approach. The results showed a K = 0.699, coming from ρ_o_ = 0.763 ρ_e_ = 0.231, which can be considered as very good and better than for the former approach.

To compare both approaches we also calculated the MAE and the MAE min (in years) retraining the network with the same number of training/test films (40/160, 80/120, 120/80, 160/40) as in the approach 1. The results can be found in Table 7.

**Table 7:** Production year prediction results using an artificial neural network.

Number of training/test films	40/160	80/120	120/80	160/40
MAE mean	11.12	9.32	8.96	8.41
MAE min	8.77	7.04	6.74	6.30

The results show a clear advantage of this approach in terms of MAE (in years) and Kappa statistic, compared to the approach 1 presented.

#### Test 3A: Genre classification, approach 1 with classical machine learning

Similarly to the year prediction test, the genre prediction test has been performed in a supervised scenario, using several films for training and several films for testing.

In the approach 1, we are using only low-level video descriptors and Support Vector Machines (SVM) as learning method.

Our first approach was to use our descriptors to classify films into three classes which have been selected for genre classification, following David Bordwell's analysis of classic genres and its analogies [26]:

1. Comedy/drama films
2. Thriller/action/horror films
3. Independent films.

The methodology of the genre classification test is divided in three phases: creation of the ground truth, training and testing.

In the first stage, each one of the 200 films of the data set was manually classified with the label of its dominant genre. In contemporary films it is not possible to classify films into a single genre, because the postmodernism paradigm has changed the classical idea of genres and nowadays multiple genres can appear in a single movie, as has been underlined by several authors [38] [39]. A dominant genre can be established for most of the movies, but it is impossible for some of them, which are labeled as independent movies.

Similarly, genre classification of classic movies is different from that of postmodern movies (for example, the characteristics of a comedy of the 30’s and a movie of the 90’s are quite different from each other). The heterogeneity of our set of films is a key factor when testing final results, because a wider range of aesthetic styles will be covered.

A previous research on this issue was developed by Rasheed *et al*. [40], who propose a framework for the classification of films into genres, based only on computable visual cues. They use only four descriptors, which are a subset of our 24 descriptor set. However, there is an important difference, because they use a data set consisting only of contemporary films (postmodernist films). Their results with that set of movies are quite good, but the framework fails when it has to deal with a more complex data set such as ours, which has movies from every age and style. This is because the features of the genres have changed along the History, and, for instance, the pace of an action movie of the 40s will be similar to a comedy or drama movie of the 90s.

Rasheed *et al*. [40] use a mean-shift clustering algorithm for the classification, which presents a lot of problems with our data set because of its diversity and does not actually yield valuable results. Therefore, we decided to use a supervised algorithm taking into account our film theory- based model, and the tests were made with a support vector machine, which was modeled using the least-squares technique (LS-SVM [41]). A multi-SVM implementation has been used.

Four different sub-tests were performed. They are described below, and results are shown in Table 8.

a) Rasheed descriptors: This test was developed using the four Rasheed *et al*. [40] descriptors: average shot length, color variance, motion content and lighting key. The normalized descriptors are directly introduced in the SVM.
b) Our descriptors: This test is similar to the first test, but it uses the complete set of 24 descriptors. However, results show no significant improvement.

The main problem of tests a and b is that they look for common genre patterns among a set of films that are too heterogeneous. Therefore, an intermediate stage is included. This stage is the aesthetic style clustering, which is described in sub-section” High-level characteristics prediction” of this paper.

**Fig 6:**
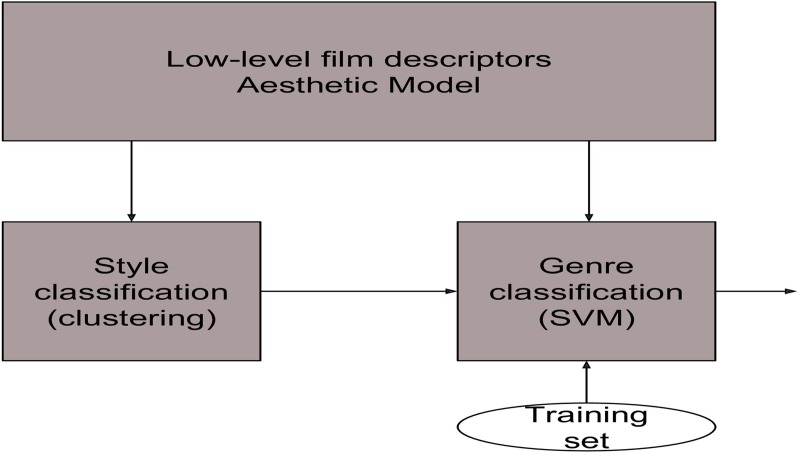
Complete genre classification technique (our implementation with styles).

c) Our implementation with styles: This test is the implementation of the aforementioned method, and it is depicted in Fig 6. The output of the style classification clustering module may be one of five possible styles, but they are grouped into three labels: Classical Films (when the output is ‘Silent Films’ or ‘Classic Films’), modern and neoclassic films (for ‘Modern Films’ or ‘Neoclassic Films’) and Postmodern Films. An independent Multi-SVM algorithm is applied to each group and the results of the classification are significantly better than with the previous tests.
d) Ground truth styles: It is the same test as the latter, replacing the aesthetic style clustering stage by a manual classification of the movies into 3 categories: classical, modern and postmodern. The results are slightly better than the complete c test, due to the errors in the clustering stage.

The results in Table 8 are obtained using a training data set of 140 films, and a test set of the remaining 60 films. Results for a smaller training set are not significant, because some of the style clusters have very few films. For a larger training set, the results remain almost constant.

**Table 8:** Genre classification results.

Test	Precision	Recall
Rasheed descriptors	0.46	0.41
Our descriptors	0.47	0.44
Our implementation with styles	0.70	0.56
Ground truth styles	0.71	0.61

From the results of these tests, it can be concluded that the visual patterns of genres are strongly linked to the cinematographic paradigm which a film belongs to.

#### Test 3B: Genre classification, approach 2 combining descriptors and image features with deep learning

For this approach, we combined the low-level descriptors extracted from films with the image (frames) analysis using deep learning techniques, in this case, Convolutional Neural Networks, based on the well-known ResNet architecture[42].

This will support the comparison with similar approaches in the literature [10–15]. In order to have a direct manner to be compared with existing approaches, we have employed in this approach, apart from our 200 films dataset, to allow a direct comparison with such works the dataset released in [15] (with name Large Movie Training Dataset). This dataset is composed of 2875 films, classified into 9 classes and providing additional valuable information: year of production, starring… among others. To apply our method, for this particular test we added the extracted descriptors presented (used for our own 200 films dataset) but using the LMTD trailers available, to allow the comparison with other works. Employing the entire films is very costly in terms of computing resources when following this approach.

There exist multiple works proposed for genre classification using artificial neural networks (ANNs) as described in section “related work”. The most similar work is presented in [11] where audio and video (image) sequences are split and ANN backbones are applied separately to extract the features. In this work, it is indicated the advantages and better performance of results using CNNs with temporal factors compared to other deep learning techniques only based on temporal factors such as LSTM.

We used a the same genres as in the papers indicated, to allow the comparison, when using the LMTD dataset. For our own dataset we are using common genres as in IMDb [43] (following the Bordwell classification). Some films were labelled with several genres (up to 3 genres).

**Fig 7:**
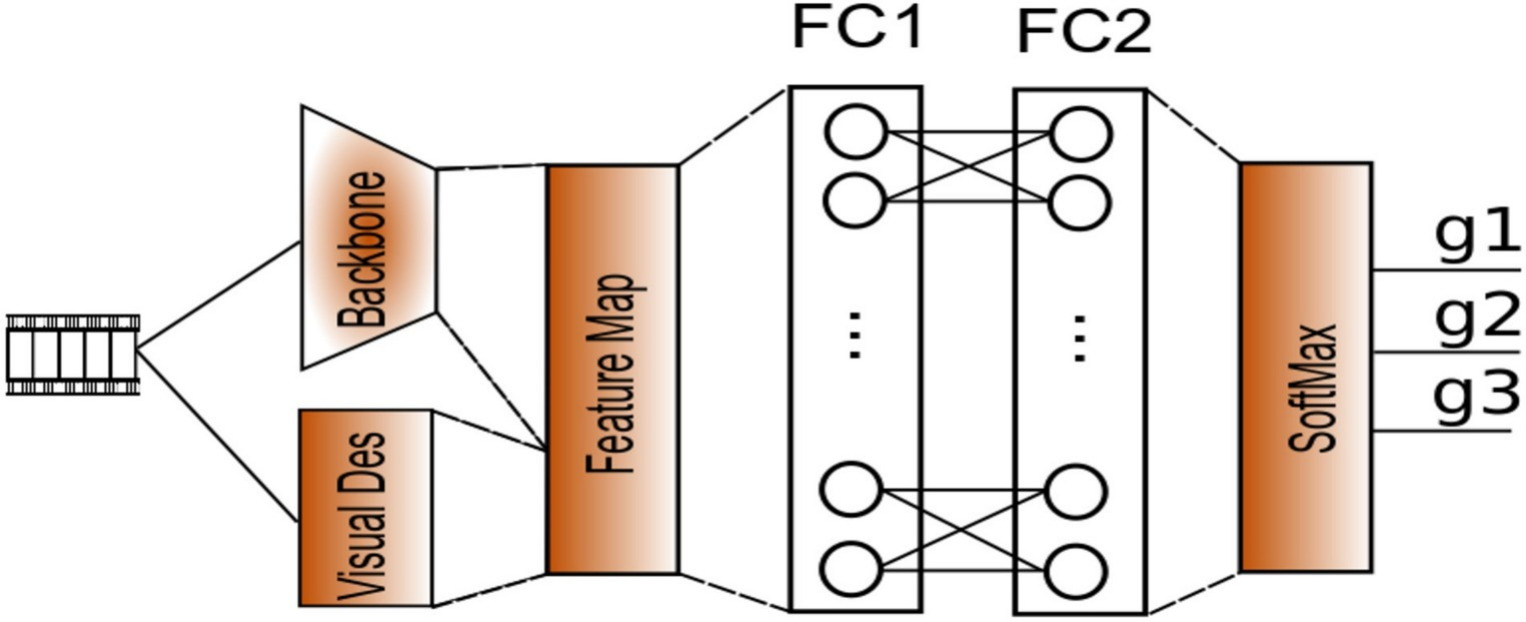
Neural network architecture for genre classification using visual descriptor and image descriptors.

**Fig 8:**
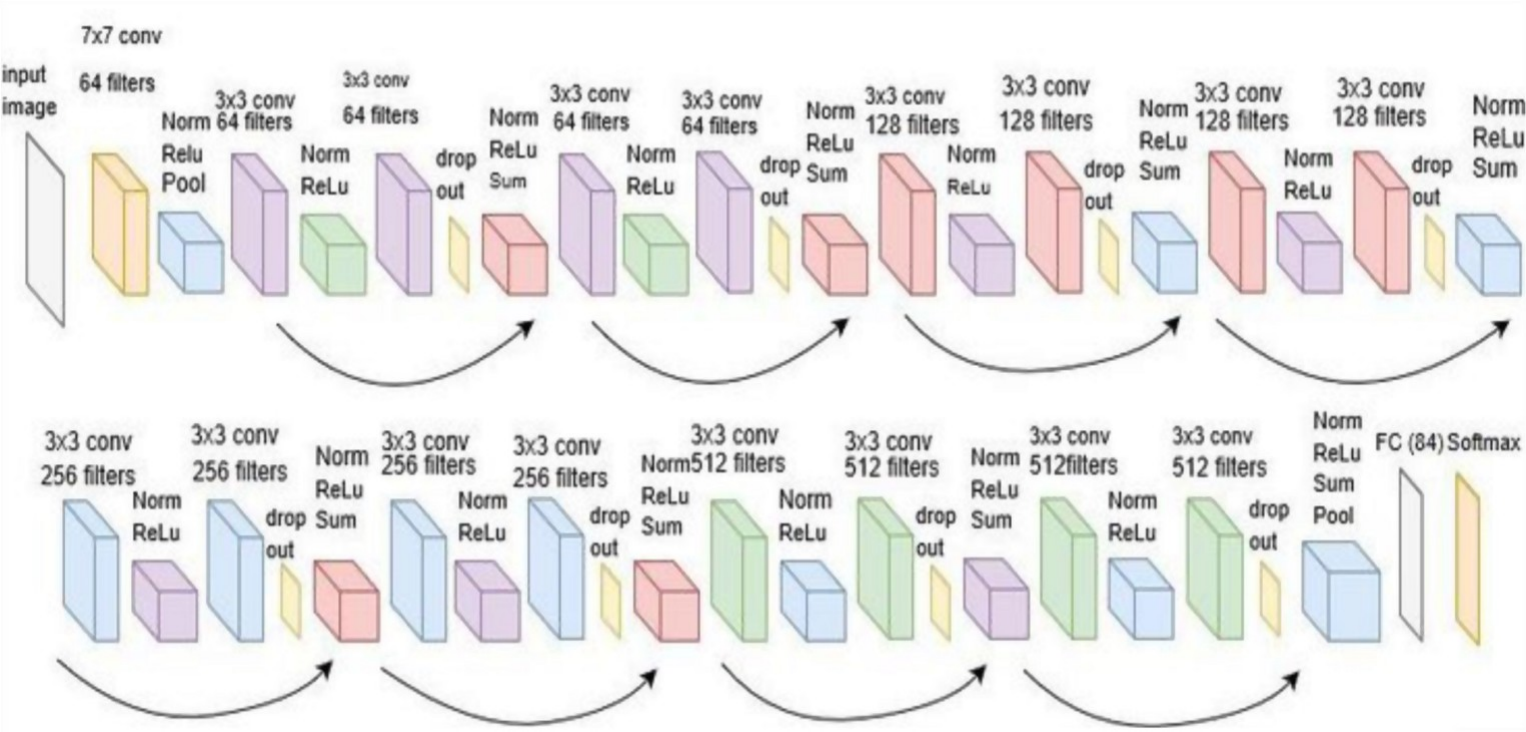
General architecture of the ResNET NN. In this work the last layer is removed to use a pre-trained model.

The architecture of the proposed neural network is depicted in Fig 7. with the general architecture of a ResNET in Fig 8. The methodology followed in this approach is based on three steps:

- Define the set of features
- Pre-process the data and extract the most relevant features
- Train the model

The set of features was defined as follows, and offered as supplemental material of this paper:

**Descriptors:** contains the aforementioned set of descriptors illustrated in *Film Characteristics and Descriptors model*.

**Visual Descriptors:** LMTD information from trailers, as well as a database containing the movies keyframes.

**Audio Descriptors:** *(optional)*, in order to allow further work beyond the visual information.

The proposed approach has been tested in both our 200 films dataset as well as literature work (LMTD) dataset to compare the performance of our model.

We started with our own dataset. To extract the results of the test, the Neural Network proposed is composed of two Fully Connected (FC) layers, followed for a SoftMax layer to make the final decision on genre. The set up can be found in Table 9 and results in Table 10.

**Table 9:** Genre classification details using ANN for the proposed 200 movies dataset.

Genre	Training	Validation
Crime	40	10
Thriller	62	14
Drama	16	5
Comedy	19	4
horror	11	5
Adventure	12	3
Romance	11	3
Sci-Fi	11	4
Biography	9	3

**Table 10:** Genre classification results, with deep learning.

Genre	Precision	Recall
Crime	0.96	0.75
Thriller	0.93	0.7
Drama	0.95	0.7
Comedy	0.96	0.72
Horror	0.89	0.75
Adventure	0.85	0.7
Romance	0.94	0.72
Sci-fi	0.93	0.76
Biography	0.79	0.73

The experiment was run employing 80% of the dataset for training and 10% for testing and validation respectively. The labeled one-hot vector for genre representation for every film (up to three labels per film) and a total size 13 genres, where due to reduced number of films in some categories, we presented the results for 9 genres. The training for the total feature vector was done with 50 epochs. As multiple labels were assigned to the same movie, then only the most probable outcomes were chosen by applying a SoftMax layer on top of the 2 layers architecture.

These results overcome the results presented in the literature, but this is mainly due to 2 factors: on the one side, the number of samples considered in this work is lesser (N = 200); on the other hand, the number of features in this work includes the visual descriptors, which implies a larger number of information in the training phase.

To allow comparison with other works, we performed this approach using Labelled Movies Trailer Dataset (LMTD) to compare performance.

The descriptors described in Table 1 were extracted for the set of trailers provided in LMTD dataset. Additionally, we extract the most relevant visual features from trailers, the complete list is detailed in [11] and is available for method comparison. We have followed the genre approach presented in [11] to allow comparison, where 9 categories for genre were established:

0. Action
1. Adventure
2. Comedy
3. Crime
4. Drama
5. Horror
6. Romance
7. SciFi
8. Thriller

**Fig 9:**
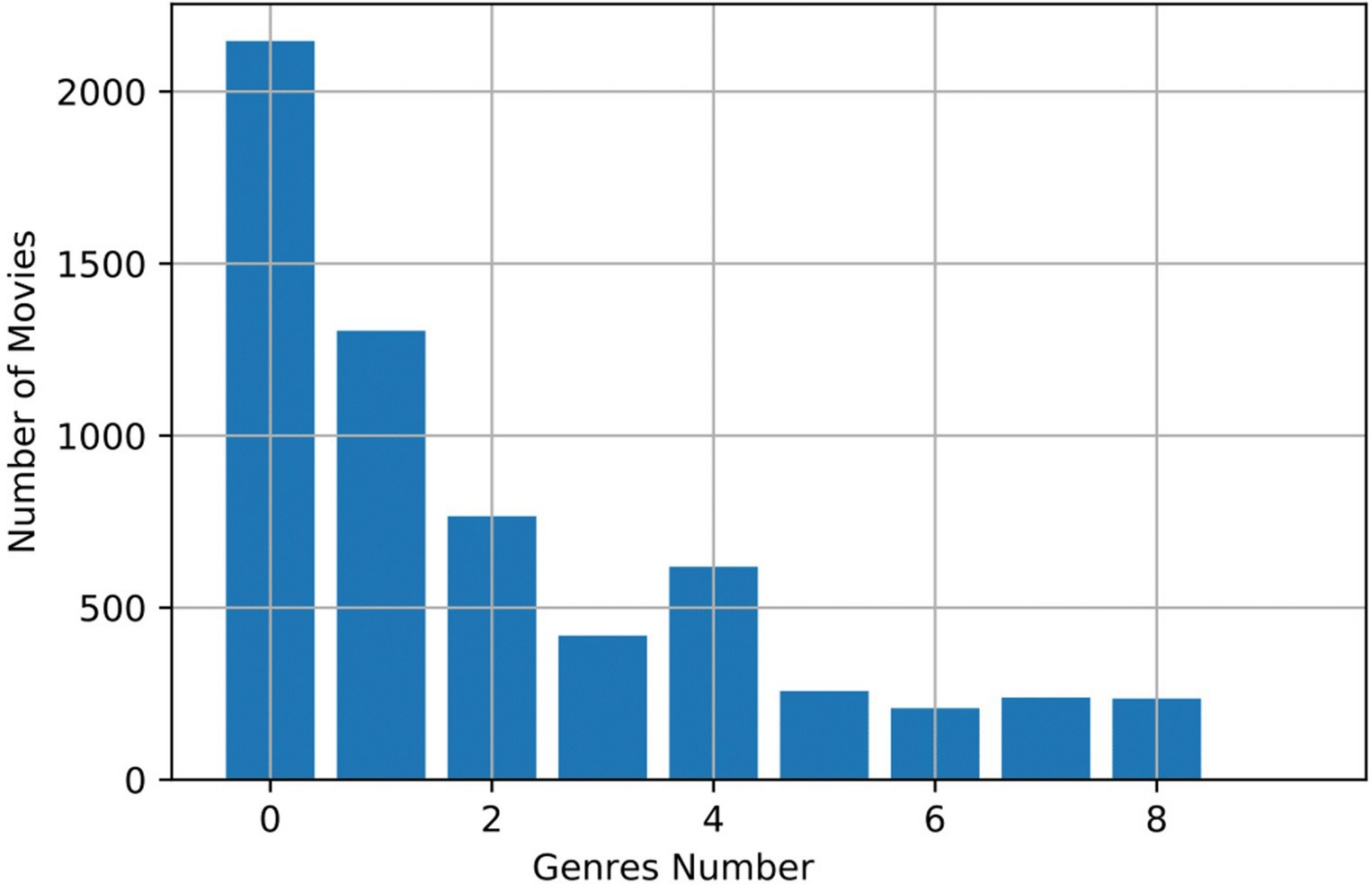
Films (movies) distribution according to its genre. Genre follow the aforementioned list and were proposed in [11].

**Fig 10:**
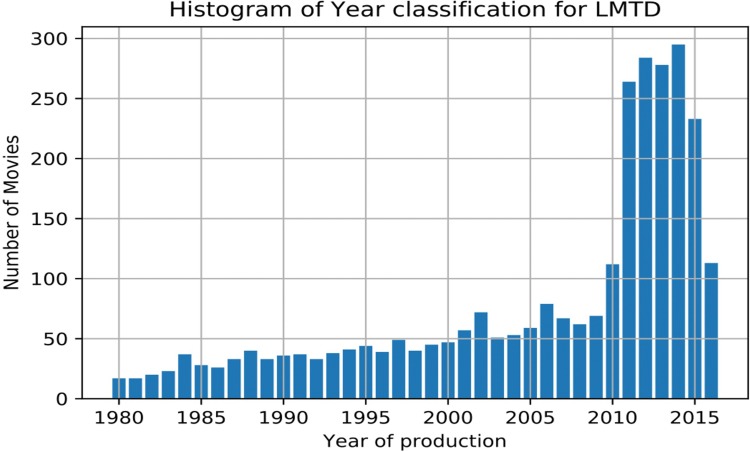
Films (movies) distribution according to its year of production. This distribution follows the aforementioned list and were proposed in [11].

The films distribution per genre is depicted in the Fig 9:

Moreover, year of production of LMTD dataset movies is in the range from 1980 to 2016, with the following distribution shown in Fig 10:

Figs 9 and 10 show that it is necessary to weight the input data for unbiased classification, as the number of inputs is not balanced. As an example, it is not equal to create a classification model for a dataset that contains only 37 movies produced in 1980 that a model that has 285 movies as input (i.e. production in 2015). The information of all sources is normalized individually (depending on the source Audio, Images or Visual Descriptors) as well as normalized when multiple-sources are available.

We have used a set of visual features to increase the information available for genre classification. More specifically, we employ the RESNET-152 as backbone which is illustrated in Fig 8, by removing the last layer (AVGPOOLING). The images are inserted into this backbone and until the last Fully Connected (FC) layer. The resulting feature vector is of 512-elements length. As employing the entire frames set is very extensive in terms of computational complexity, the main statistics on the information are used. These statistics include the mean, standard deviation, skewness and median of the movies feature maps. Therefore, the set of visual descriptors shown in Table 1 are considered in our tests, which represents an added set of features that are not considered in the related work. Then it is optional to include audio and feature maps from the trailers, which result in the construction of a 3030 length feature vector which is inserted into the backbone classifier.

Although in this work it was not considered, audio were added to our dataset for further research purposes, not used in our training process as it is out of scope. The large dataset provided as supplemental material contains a total number of 2981 numerical features as well as 6 categorical variables, in addition to the code of the proposed methods and some pre-processing utilities to extend the scope of this work can be found at: https://gitlab.com/tavitto16/Movies_analysis

First, we analyzed the influence of the descriptors only proposed in this work in Table 1 and its impact on the quality of estimations. We have inserted this information for each of the 2871 films in the training set. The model was trained using a Stochastic Gradient Descent SGD optimizer with Learning Rate and Beta parameters of 0.001 & 0.002 respectively. Furthermore, the entire dataset was split into 64 elements batch size and trained for 30, 50 and 100 epochs respectively. The accuracy of the model is not significant, reaching a mean value of 60%.

However, its impact on the quality of estimation is high, as the number of features is limited. We proposed to combine this information with visual content of the frames to increase the information available in the classification procedure. The experiments performed in this paper were executed on a CPU Intel Core i9-7900X at 3.3GHz, 64 GB RAM DDR5 and a GPU NVIDIA Pascal GeForce GTX1080 Ti. The execution time was:

- 144.44 Seconds for 100 epochs and 64 Batch size
- 72.532 Seconds for 50 epochs and 64 Batch Size.
- 44.849 seconds for 30 epochs and 64 Batch size.

(when descriptors are already extracted from content).

Second, we have analyzed the data extracted from the frames available in [11] to combine them with the descriptors. As this work is the most similar to the work presented, we compare with this approach by employing the Keyframes provided. We have obtained the visual features for every item in the dataset by inserting the frames into the predefined Backbone. In [11], a pre-trained model for a RESNET152 architecture is provided and it has been used in the classifier training process. The images are flattened and cropped according to RESNET152 backbone input. The output vector is 1024-length features, which are normalized to be non-negative and are appended to the descriptors previously described to create a Feature Map of 1050 features.

The optimizer and its corresponding parameters Learning Rate and Beta remain in 0.001 and 0.002. The training process is performed 30, 50 and 100 epochs respectively. Results of main metrics are shown in Table 11.

**Table 11:** Genre classification using Deep Learning for LMTD movies dataset.

	Accuracy	Precision	Recall
**Action**	0.96	0.91	0.75
**Adventure**	0.95	0.89	0.7
**Comedy**	0.94	0.85	0.7
**Crime**	0.92	0.81	0.72
**Drama**	0.89	0.79	0.75
**Horror**	0.85	0.77	0.7
**Romance**	0.64	0.73	0.72
**SciFi**	0.72	0.74	0.68
**Thriller**	0.71	0.73	0.69

The execution time for the experiments performed was of 1h 21 mins and 22 seconds. Furthermore, the LMTD provided the train dataset, being the test and validation subsets able only under request. Therefore, we have split our dataset into addition to the subset created by 2012 films for training and a 431 testing items and 431 for validation respectively.

In order to compare with other works, we took the best results found in the literature as far as we are concerned, which are found in [11]. The results are compared to the ones obtained in [11] in the Table 12, using the same metrics, in this case the Area Under the Curve:

**Table 12:** Genre classification comparison of the proposal method with existing approaches, measured with the AUC (Area Under the Curve) metric as in [11].

	Our Method	LSTM [11]	CTT-MMC-TN [11]
Action	**0.852**	0.687	0.835
Adventure	**0.752**	0.573	0.672
Comedy	**0.871**	0.792	**0.870**
Crime	**0.628**	0.421	0.547
Drama	0.641	0.740	**0.841**
Horror	0.424	0.478	**0.667**
Romance	**0.468**	0.313	0.456
SciFi	0.192	0.237	**0.401**
Thriller	**0.520**	0.437	**0.522**
Weighted Mean (per number of elements) of AUC results	0.762	0.638	0.760
Arithmetic Mean of AUC results	0.594	0.520	0.645
Standard Deviation of AUC results	0.218	0.192	0.175

In this table, our work is compared with the same metric and dataset against a Long Short Term Memory (LSTM) and a Convolution-Through-Time for Multi-label Movie genre Classification (CTT-MMC) approaches, as described in [11].

From these results, it can be observed that the proposed algorithm outperforms the AUC obtained in the related works for some particular genres, especially those with many available films per category. It is probably due to the fact that the number of samples for these particular methods (Action, Adventure, Crime and Comedy) is large, and that the influence of the descriptors allow the system to better define particular features for every single classes. The architecture is similar, as well as the model parameters, therefore, it can be argued that the enhancement could be reached by the information added by the descriptors presented in this work. However, the results are rather poor for Thriller and SciFi genres, mainly because of the very few items available in the dataset (see Fig 9).

The unbalanced dataset problem can be faced in several manners: balancing the dataset by appending new items, as currently there is a wide range of open sources with data labelled for all particular genres, or employing resampling methods [44] such as boostrap. The former consists in performing multiple iterations taking “small balanced subsets” that allow the system to better understand the features of all classes. Bootstrap technique cannot be directly applied over the dataset as it requires data augmentation from the existing dataset, which requires generative models for image/video data creation (learning styles). In practice, with a reduced amount of items appended to the less common methods, it is possible to employ this technique to define confidence intervals and mitigate the uncertainty of such rare classes, which can enhance the results achieved. Another approach, such as weighted resampling is not feasible to be applied in the dataset as genre is in a fuzzy classification problem.

**Fig 11:**
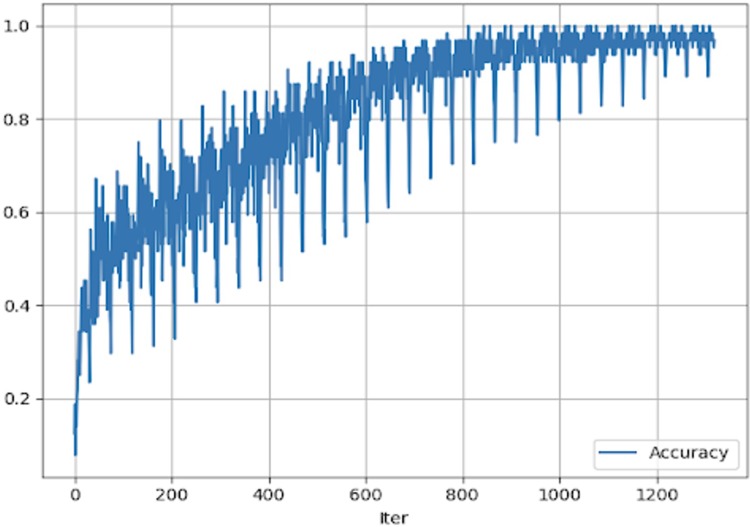
Accuracy evolution, using all relevant visual, descriptors and audio features for 50 epochs.

Finally, although it is out of the scope of this paper, we check the possibility to add audio features and they were appended to the estimation process, similarly to the literature work [11] attaining a slight improvement as shown in Fig 11

#### Test 4: Popularity prediction

The popularity prediction test has been developed in a similar way to the previous case, using popularity data extracted from the website IMDb [43], which is perhaps the most used film database, with the biggest number of ratings per film. We have computed the popularity of a film as: Popularity = Number of ratings of the film * Average rating of the film, where the number of ratings and the average rating of the film are public data, available in IMDb. Using this expression, we combine the familiarity and favorable opinions; both are usually used to obtain several popularity measures, such as Q-score or Q-rating. Other approaches to movie popularity take into account only the average IMDb rating of the film [45][46] (with some additional restrictions, such as the decade or the minimum number of ratings), but we use very diverse films, from every decade; therefore, we have to weight this value with the measure of the number of ratings.

The implemented classification technique is analogous to the one used for production year prediction. However, a different set of base descriptors has been selected, after calculating the most influential parameters according to a PCA. The 10 used descriptors have these Ids: 1, 6, 7, 8, 10, 14, 15, 17, 23 and 24.

**Fig 12:**
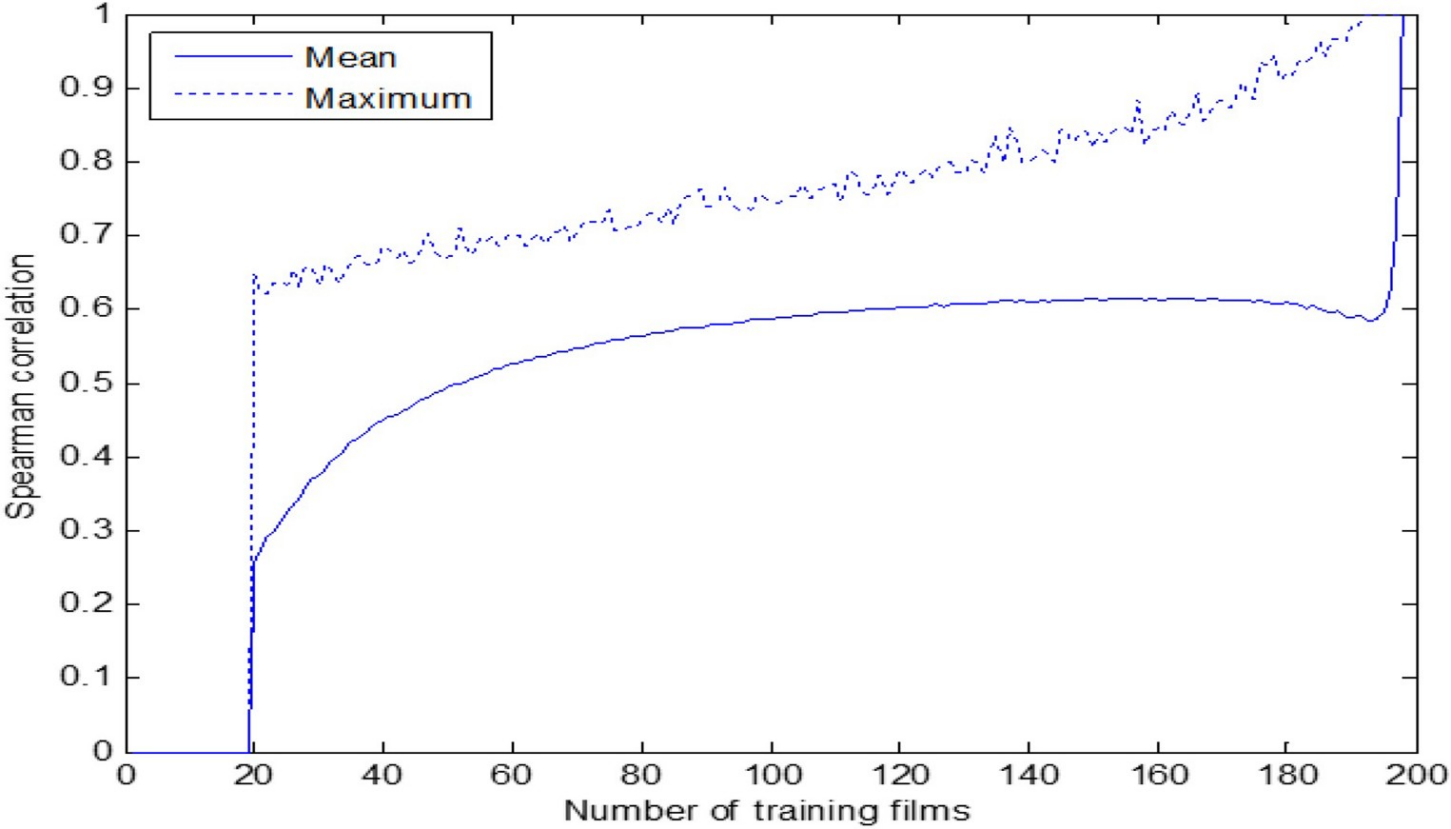
Spearman correlation of popularity prediction.

The developed tests are also analogous to the production year prediction test, and the results are shown in Fig 12 and Table 13. In this case, the objective is to arrange the test set of films in a descending order according to the predicted popularity. The similarity between the predicted and the real list is measured by means of the Spearman correlation.

**Table 13:** Popularity prediction results.

Number of training/test films	100/100	120/80	140/60	160/40
Cor. Spearman mean (k = 10000)	0.5872	0.6022	0.6123	0.6164
Cor. Spearman max	0.7693	0.8039	0.8046	0.8797

The results show that our aesthetic model has an influence in the estimation the popularity of a film, and which are the most influential descriptors for such purpose. The differences between the average results (spearman) and the best results (spearman max) are due to the selection of the training set: first one is random selection, and the second is with carefully chosen representative success films from different periods.

## Conclusion

Film aesthetic modeling had not yet been considered to create real applications related to video recommendation, Decision Support Systems (DSS), or any other predictive application. It had only been taken into account manually by film analyzers, but there were no models to implement automatic real systems. The existing automatic systems only use other kind of information, related to features such as director, actors, nationality or collaborative techniques.

In this paper, we have presented an automatic aesthetic modeling based only on low-level visual features and we have proved its effectiveness in supervised and unsupervised scenarios. We have taken real films and have presented results of our own complete implementation of the system core. We have proposed 4 applications out of the many that can be developed using this core, and we combined our low-level descriptors model with other image analysis techniques based on deep learning such as convolutional neural networks to obtain increased results.

We have expanded the set of descriptors with respect to Wang's [3] and Canini's [2] approaches. This allows us to apply the model not only to isolated scenes of films, but also to entire films. We do not classify spectator emotions into predefined categories (cold/warm, slow/dynamic, etc.) as we take into account directly a multidimensional approach, which makes our system more flexible and adaptable to several applications. We have proposed an unsupervised method to classify films, and developed a tool that automates, enriches and enhances the aesthetic annotation processes of other existing tools (Cinemetrics [1]) which are well-known in the film academic sphere. Our model has also been tested using supervised prediction algorithms, and has proved its outstanding performance when compared to other approaches. Last but not least, we have proved the importance of aesthetics in several applications regarding film classification, which opens a huge range of possibilities. We have shown that it is possible to classify films (for example, regarding year prediction or genre detection) using only low-level visual features, if we take into account film theory in order to apply aesthetic aspects to model the data and create methodologies; and that our low-level video descriptors can have an influence in the prediction of the film popularity. This has been shown especially in the case of genre classification, because a comparison between a method with no aesthetic considerations and another taking into account these characteristics has been performed. The combination of deep learning models such as CNNs for image (frames) analysis and our descriptors model showed a great promise in genre classification. The information from images (visual features) was used to increase the visual information in both supervised and unsupervised classification methods. As future work, additional temporary information derived from the intra-frames can be employed to extract sequential descriptors that could support the estimation of complex film features such as activity-based, action type classification, and use a higher number of films.

The hybridization of this aesthetic model with other semantic, collaborative or statistical approaches, along with audio descriptors, can open up new lines of research.

**Fig 13:**
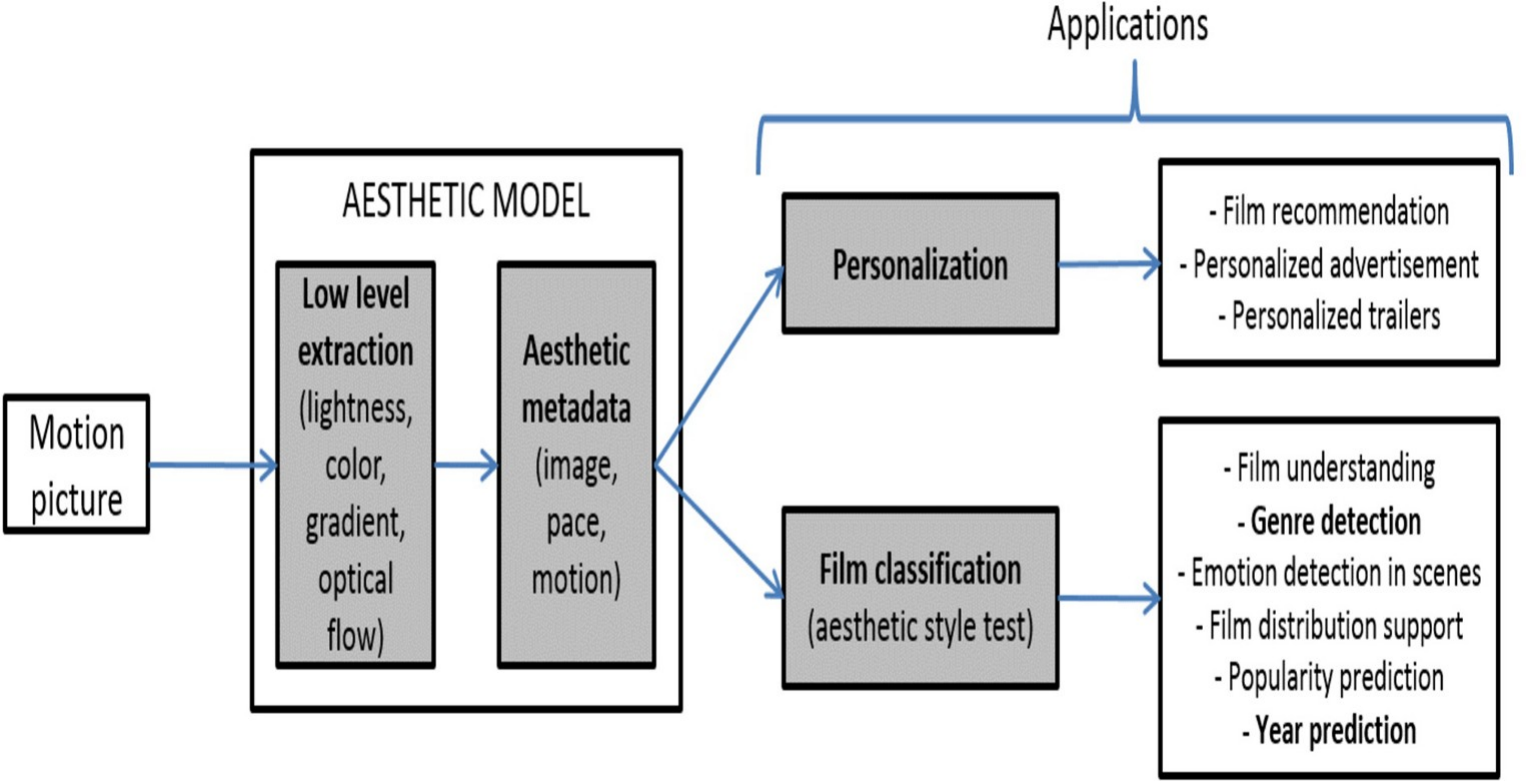
Proposed aesthetic model and possible applications.

These lines are related to video customization (for example, automatic generation of videos aesthetically pleasant to each user), video recommendation, video analysis (for example, for film theorists) or prediction of different features, with several options for film producers, distributors or companies which develop audiovisual services. A summary of these lines can be found in Fig 13

## Supporting information

S1 File Appendix. Automatic Descriptors Extraction.
(DOCX)

S2 File Dataset of audio, image and descriptors for movie trailers. (CSV)

S3 File LMTD audiovisual features and descriptors processed to allow Deep Learning approach. (ZIP)

S4 File Movies features dataset used in the experiments. (CSV)

S1 Fig (TIFF)
