# Supplementary material for: On the influence of low-level visual features in film classification
Source: PLoS One. 2019 Feb 22;14(2):e0211406. doi: 10.1371/journal.pone.0211406 (PMC6386315; doi:10.1371/journal.pone.0211406)
Supplement: S1 File — Automatic Descriptors Extraction. (DOCX) [file pone.0211406.s001.docx]

# S1 Appendix: Automatic Descriptors Extraction

Recent developments on audiovisual low-level descriptors analysis have led to great progress in video characterization tasks. Video annotation practices have improved remarkably due to the emergence of new techniques, such as multi-labeling over shot sequences by incorporating spatial and temporal context [1], as opposed to video annotation paradigms working on individual shots. The well-known gap between low- and high- level features is becoming ever narrower due to innovative fusion techniques, which take into account semantic patterns along with visual and speech features [2]. Another traditional limitation is the domain dependence, which causes problems related to insufficiency of training data and inefficacy for large-scale video datasets. In [3], a novel approach is proposed based on semi-supervised learning by using information from the Internet for interesting event annotation in multi-domain videos. One recent domain where these techniques are being applied is Smart TV (for social videos). In [4], an automatic video annotation technique is proposed, employing ontologies which describe the contents of a video to facilitate video retrieval.

These advances have allowed a consequent development of the applications exploiting data from video annotation techniques, such as summarization [5], retrieval [6][7] or segmentation. This is an essential component of video characterization, as it allows establishing remarkable differences inside the sequences of a video, providing easier and more precise classification. According to Megret [8], video segmentation methods are classified into three types: segmentation with spatial priority, segmentation with temporal priority, and joint spatial and temporal segmentation. On the one hand, pure spatial priority is related to the analysis of still images from key-frames or recognized objects. On the other hand, pure temporal priority is related to motion analysis, a technique which has been developed and exploited in recent years. For instance, in [9], video segmentation is implemented by means of motion coherence analysis. Using a tracking process based on adaptively sampled points, 2-D motion patterns are identified with an ensemble clustering approach. These points are clustered to obtain a pixel-wise segmentation in space and time domains. Each cluster represents a different area of the scene that moves coherently along the video sequence. The clustering result is mapped into an image spatial-temporal feature space.

Moreover, several algorithms have been exploited to segment videos by motion analysis. For example, the QuickShift segmentation algorithm is used in [10] to segment the video frames into different regions based on motion and appearance cues. In [11], Zernike moments are computed in directional motion history images, which are an extension of the optical flow method for object tracking, and pair-wise pixel comparison is carried out to perform temporal segmentation of isolated utterances.

The descriptor model can be as flexible as a film theorist may want but it should be implementable as an automatic tool, using video processing techniques. This automatic tool will allow for the validation of the model and the creation of different applications for film analysis, high-level feature prediction, etc. Therefore, an essential requirement in our model is that every defined descriptor can be automatically extracted from a video. This extraction should be efficient enough to process the video in real time if needed.

The accurate selection of image descriptors depends critically on two tasks: the right selection of key frames, and the correct detection of shots. The second one is also crucial to perform an accurate pace modeling, as it is based on video shot segmentation.

1. Optical flow

The Lucas-Kanade algorithm [12] is used to obtain the value of the optical flow. It is applied to a set of points established in the image and homogeneously distributed: there are 8 external points and 9 internal points. These two kinds of points are analyzed to establish differences between camera movement and internal movement. A threshold (for comparing the values of external and internal points) is used to infer when the camera is moving and the described motion metrics are directly applied to the video.

1. Temporal segmentation

According to the state of the art and the available information (optical flow), an algorithm that extracts cuts with very high precision has been designed. The strategy adopted is described below:

- The harmonic mean of the motion vectors of each frame is calculated, and subtracted to the same value of the last frame (this prevents confusion with the camera motion). As a normalized value is obtained, a threshold is enough to decide when there is a cut. The harmonic mean is used to lower the relevance of high values, which may come from the abrupt movement of an object in a scene. Using this technique, precision and recall are very high, although it needs some refinement: we set a low threshold which gets maximum recall, but not very high precision, and we introduce a second step.

- The second step is a measure of correlation between three frames before the cut and three frames after the cut. These six frames are a security margin to avoid synchronization failures. The correlation metric is obtained from [13] and described in Eq. A, where *x_ij_* and *y_ij_* are the pixels of the first and second images, respectively.

$c=\frac{\sigma_{xy}}{\sqrt{\sigma_{xx}\cdot\sigma_{xy}}}=\frac{\sum_{i=1}^{n} \sum_{j=1}^{n} \left( x_{ij}-\bar{x} \right)\cdot\left( y_{ij}-\bar{y} \right)}{\sqrt{\left( \sum_{i=1}^{n} \sum_{j=1}^{n} \left( x_{ij}-\bar{x} \right)^{2} \right)\cdot\left( \sum_{i=1}^{n} \sum_{j=1}^{n} \left( y_{ij}-\bar{y} \right)^{2} \right)}}$ (A)

This operation is used to rule out false positives and requires extra processing but the system is still capable of processing in real time.

Other possible transitions between shots are the dissolve, and fade in or fade out. It is important to distinguish them from cuts, because both carry other information peculiarities: for example, they are used to change the narrative pace, to change from a sequence to the next, or to create narrative ellipses. We use a measure of the gradient of the candidate images (selected by an anomalous optical flow), to detect them. Fades are easy to detect, because the image turns black (or any other color), and edges disappear: in this case the gradient is null. Dissolves are detected because the number of edges presents an increasing monotone function until the second shot gets the same intensity as the first shot (two superimposed images means double number of edges), and a decreasing monotone function until the shot gets stabilized. The implementation of this technique is based on Chen Xu *et al.* approach [14].

Another important issue is scene detection, but only an approximation can be reached, since scene segmentation also depends on narrative issues, which are not taken into account. However, a good qualitative approach is obtained using the scene detection by shot grouping algorithm from Lin and Zhang [15], to which we add some narrative constraints (such as detecting insert shots by adding entropy features to the comparison of the correlation between non-consecutive shots).

A summary of the extraction model, linking with the descriptors, can be found in the Fig. S1

S1 Fig. Global descriptors extraction model.

1. Key frame selection

A first approach to key-frame selection was made by processing every frame of a shot. However, it involves a lot of computational load, and it was discarded. Since we only need to detect the kind of shot (to obtain image features and detect scenes), one key frame is needed for each shot. The implemented procedure is described below.

Firstly, 3 frames of the shot are selected in a pseudo-random way, by fixing a single constraint: a minimum time T must exist between the selected frames, as well as between them and the beginning or end of the shot. in our implementation, *T* is estimated as 10% of the total duration of the shot.

The 3 selected frames are compared. If they are similar, any of them is chosen; otherwise, the less similar frame is replaced by another random frame until a similarity condition is reached. In the worst-case scenario, when the condition is never met, every frame of the shot is processed.

The frames are compared using the color histogram, (which is the most sensitive parameter when the camera or the objects of the images are moving). When processing black and white films, entropy is computed instead of the color histogram.

Once these features are gathered, the computation of final descriptors is immediate. Partial results by shots and scene are stored during the processing, and final results are obtained when finished. The high-level pseudo-code of the general process is shown in Table S1.

S1 Table. Pseudo-code of the descriptors extraction process

| For frame f=1 to end |
| --- |
| Optical flow extraction. |
| Gradient measuring. |
| Calculation of motion descriptors. |
| If (optical flow & gradient) satisfy constraints |
| Kind of shot change selection. |
| Key frame selection. |
| Calculation of image descriptors. |
| Calculation of shot-related pace descriptors. |
| If (scene detection) satisfy constraints |
| Calculation of scene-related pace descriptors. |
| End If |
| End If |
| End For |
| Final statistics calculation. |

Our software has been implemented in C++, using the computer vision library OpenCV, and some metrics about efficiency have been obtained. On average, the computation of a 90-minute film in SD (encoded in H.264 at 1 Mb/s) with an ordinary PC (Intel i7) takes 60 minutes. When 5 films are processed in parallel, the processing time is about 80 minutes. If we process an HD film (also H.264 at about 5 Mb/s), the processing time for one film is about 90 minutes.

# References

1. Li Y., Tian Y., Duan L.-Y., Yang J., Huang T., Gao W.. "Sequence Multi-Labeling: A Unified Video Annotation Scheme With Spatial and Temporal Context," IEEE Transactions on Multimedia, vol.12, no.8, pp.814-828, Dec. 2010.
2. Tseng V.S., Su J.-H., Huang J.-H., Chen C.-J.. "Integrated Mining of Visual Features, Speech Features, and Frequent Patterns for Semantic Video Annotation". IEEE Transactions on Multimedia, vol.10, no.2, pp.260,267, Feb. 2008.
3. Zhang T., Xu C., Zhu G., Liu S., Lu H.. "A Generic Framework for Video Annotation via Semi-Supervised Learning," IEEE Transactions on Multimedia,, vol.14, no.4, pp.1206,1219, Aug. 2012.
4. Jeong J.-W. Hong H.-K. and Lee D.-H. "Ontology-based automatic video annotation technique in smart TV environment," IEEE Transactions on Consumer Electronics, vol.57, no.4, pp.1830,1836, Nov. 2011.

Peng J, Xiao-Lin Q. K”eyframe-based video summary using visual attention clues”. IEEE MultiMedia. 2009 Sep 10(2):64-73.

1. Ejaz N., Mehmood I. and Baik S., “Efficient visual attention based framework for extracting key frames from videos”, Signal Processing: Image Communication, Oct. 2012.
2. Hu W., Xie N., Li L., Zeng X. and Maybank S. "A Survey on Visual Content-Based Video Indexing and Retrieval," IEEE Transactions on Systems, Man, and Cybernetics, Part C: Applications and Reviews, vol.41, no.6, pp.797,819, Nov. 2011
3. Chen X., Hero A.O. and Savarese S. "Multimodal Video Indexing and Retrieval Using Directed Information". IEEE Transactions on Multimedia, vol.14, no.1, pp.3,16, Feb. 2012.
4. Megret R. and DeMenthon D. “A Survey of Spatio-Temporal Grouping Techniques”. Univ. Maryland, College Park, Tech. Rep. LAMP-TR-094, March 2002.
5. Silva L. S. and Scharcanski J. ”Video Segmentation Based on Motion Coherence of Particles in a Video Sequence”, IEEE Transactions On Image Processing, Vol. 19, No. 4, April 2010.
6. Izadinia H., Saleemi I. and Shah M. “Multimodal analysis for identification and segmentation of movingsounding objects,” IEEE Transactions on Multimedia, vol. 15, no. 2, pp. 378 –390, Feb. 2013.
7. Shaikh A.A., Kumar D.K. and Gubbi J. “Automatic visual speech segmentation and recognition using directional motion history images and Zernike moments”. The Visual Computer. 2013 Oct 1;29(10):969-82.
8. Lucas B. D. and Kanade T. “An iterative image registration technique with an application to stereo vision”. Proceedings of Imaging Understanding Workshop 2001, pages 121—130
9. Potetz B. and Lee T. S., “Statistical correlation between two-dimensional images and three-dimensional structures in natural scenes”. *Journal of the Optical Society of America A*, vol. 20, no 7, 2003.
10. Xu C. and Wei L., "Study on Shot Boundary Detection Based on Fuzzy Subset-Hood Theory," Intelligent System Design and Engineering Application (ISDEA), 2010 International Conference on , vol.2, no., pp.476,480, 13-14 Oct. 2010
11. Lin T. and Zhang H.-J. "Automatic video scene extraction by shot grouping," *Proceedings. 15th International Conference on Pattern Recognition*, vol.4, no., pp.39-42 vol.4, 2000
