# Supplementary figures and images for: On the influence of low-level visual features in film classification
Source: PLoS One. 2019 Feb 22;14(2):e0211406. doi: 10.1371/journal.pone.0211406 (PMC6386315; doi:10.1371/journal.pone.0211406)

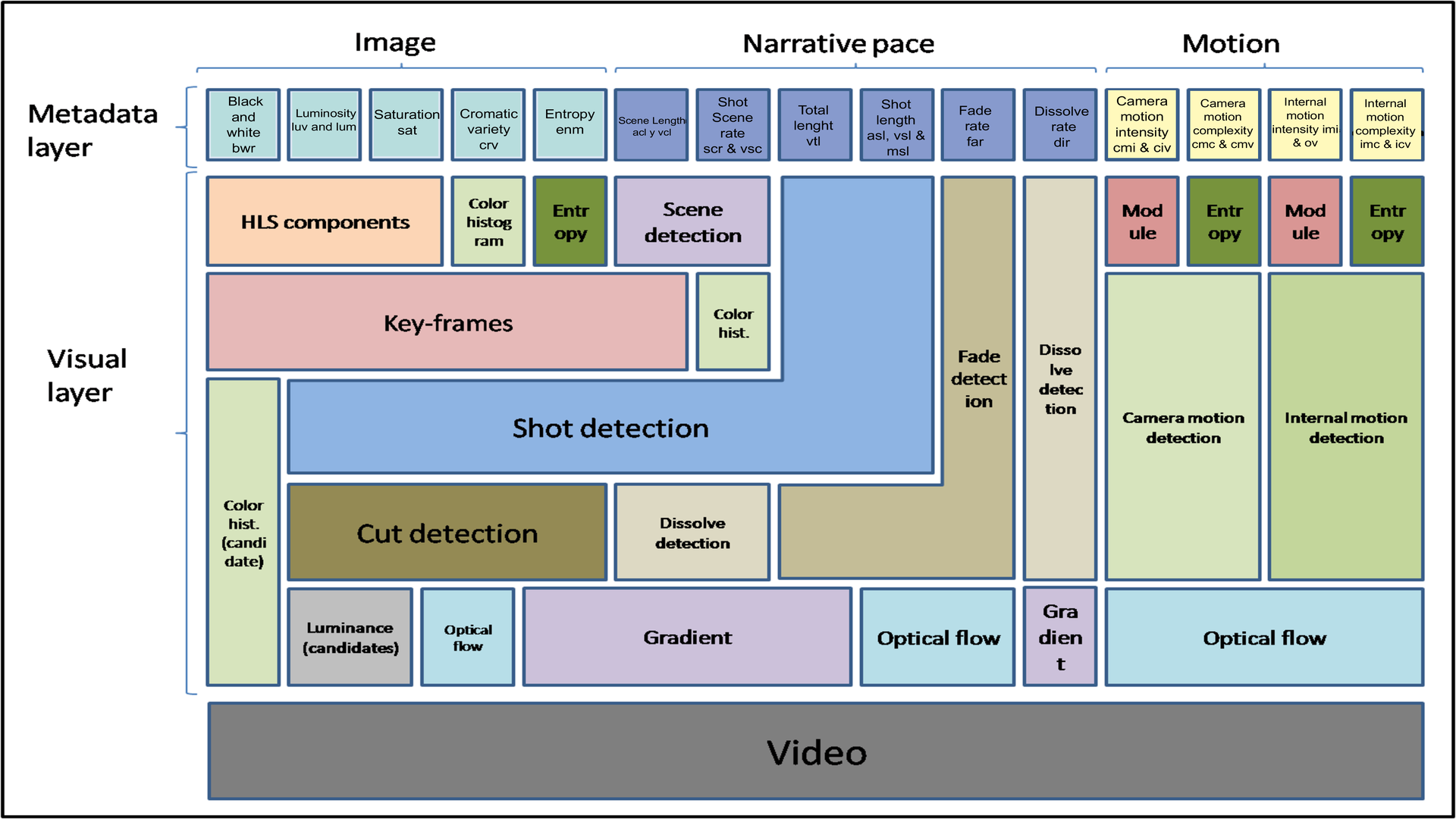

Supplement: S1 Fig — (TIFF) [file pone.0211406.s005.tiff]
